# Taxonomic review of *Ceratozamia* (Zamiaceae) in the Sierra Madre Oriental, Mexico

**DOI:** 10.3897/phytokeys.100.23152

**Published:** 2018-06-21

**Authors:** Lilí Martínez-Domínguez, Fernando Nicolalde-Morejón, Francisco Vergara-Silva, Dennis Wm. Stevenson

**Affiliations:** 1 Laboratorio de Taxonomía Integrativa, Instituto de Investigaciones Biológicas, Universidad Veracruzana, Xalapa, 91190, Veracruz, México; 2 Centro de Investigaciones Tropicales, Universidad Veracruzana, José María Morelos 44, Zona Centro, Xalapa, 91000, Veracruz, México; 3 Laboratorio de Sistemática Molecular (Jardín Botánico), Instituto de Biología, Universidad Nacional Autónoma de México, 3er Circuito Exterior, Ciudad Universitaria, Coyoacán 04510, México, D. F., México; 4 The New York Botanical Garden, Bronx, Nueva York, 10458-5120, USA

**Keywords:** Cycadales, Mexican cycads, Neotropical gymnosperms, taxonomic circumscription

## Abstract

The genus *Ceratozamia* is revised for the Sierra Madre Oriental in Mexico. This region is one of the biogeographic areas with the greatest diversity of species in this genus. These species are highly variable morphologically and this variability has led to a complex taxonomic history with many synonyms, particularly with reference to *C. mexicana*. We present a comprehensive taxonomic revision with history of nomenclature and the morphology, relationships, distribution and use of these species. We also introduce a key for their identification, descriptions, full synonymy, nomenclatural notes, etymologies and neotypes as well as taxonomic comments describing relevant taxonomic changes. We recognise fourteen species in this biogeographic province: *C. brevifrons*, *C. chamberlainii*, *C. decumbens*, *C. delucana*, *C. fuscoviridis*, *C. hildae*, *C. kuesteriana*, *C. latifolia*, *C. mexicana*, *C. morettii*, *C. sabatoi*, *C. tenuis*, *C. totonacorum* and *C. zaragozae*. This study provides a foundation for future taxonomic work in Neotropical species of *Ceratozamia*.

## Introduction

*Ceratozamia* Brongn. is one of the eight genera in the most diverse cycad family, Zamiaceae (order Cycadales). This genus is endemic to Mega-Mexico (*sensu* Rzedowski 1991), ranging from Mexico to Honduras. Mexico is considered the centre of diversity for *Ceratozamia*, given that 30 out of 31 recognised species occur in its territory (Vovides et al. 2004a; Nicolalde-Morejón et al. 2014), 90 percent of which are endemic (Calonje et al. 2013-2018). Diversity for this genus is particularly prevalent along the Sierra Madre Oriental and Southwest Mexico, especially in the political states of Veracruz, Oaxaca, Chiapas and Tabasco. *Ceratozamia* plants mainly inhabit high-elevation cloud forests (“bosque mesófilo de montaña”), pine-oak forests and relatively elevated sectors of evergreen forest in a narrow but continuous distribution (Moretti et al. 1980; Vovides et al. 2004b).

Ecologically, *Ceratozamia* requires conditions of abundant humidity to ensure proper development of immature embryos because the seeds are usually released from ovulate strobili approximately one year before germination (Norstog and Nicholls 1997). Considering the deforestation rates in Mexico and historical patterns of botanical collection, *Ceratozamia* is amongst the most threatened plant groups in the country and the genus is placed in the IUCN Red List (IUCN 2016), listed on CITES Appendix 1 and listed in the ‘Norma Oficial Mexicana NOM-059-SEMARNAT-2010’ (SEMARNAT 2010; Donaldson 2003). The latter is an official document issued by Mexican authorities listing protection categories under which diverse taxonomic groups should be placed.

Species of *Ceratozamia* are similar in morphology, particularly with regards to vegetative characters’ states, making taxonomic identification problematic. The most recent species-level taxonomic treatment of the genus is over eight decades old (Schuster 1932). Since then, researchers have focused on the study of the taxonomy of individual species and/or species complexes (Vovides et al. 2003; Osborne et al. 2006; Pérez-Farrera et al. 2009; Vovides et al. 2016).

In recent taxonomic works, quantitative morphological characters such as plant size and leaflet width have been commonly used as a basis for the identification and description of new species because they exhibit variability across the genus (Vovides et al. 2004a, c; Whitelock 2004). However, other contemporary studies have shown that these characters can be highly variable between and amongst populations, which limits species diagnosis (Martínez-Domínguez et al. 2016; 2017a, b, c). Other characters commonly used are the direction, form and texture of leaflets and the formation of prickles (Pérez-Farrera et al. 2001; Vovides et al. 2003). However, analysis of morphological patterns for these characters along the distribution range of *Ceratozamia* indicates that leaflet shape is highly similar in many species (Stevenson et al. 1986).

*Ceratozamia* was first described by Brongniart (1846) based on the sole species *C. mexicana*, which was described from wild specimens later cultivated at the Natural History Museum of Paris. Later, Miquel (1847, 1848) described five new species –namely, *C. brevifrons*, *C. intermedia*, *C. latifolia*, *C. longifolia* and *C. robusta*. In 1849, this Dutch botanist reduced *C. intermedia* as a variety of *C. longifolia* (var. minor Miq.). Later on, Regel (1857a, b) recognised only *C. mexicana* and *C. robusta*, along with a new species, *C. kuesteriana* Regel. All other Miquel names were placed in synonymy within *C. mexicana*.

Miquel (1861) recognised all species listed in his 1849 work plus *Ceratozamia kuesteriana* and subsequently synonymised all of these names with *C. mexicana* (Miquel 1868, 1869a, b). Regel (1876a, b) conserved his 1857 system, which recognised *C. robusta*. The two most recent taxonomic treatments of *Ceratozamia*, written by Thiselton-Dyer (1884) and Schuster (1932), are highly contrasting in the recognition of species and infraspecific categories. The first author recognises four species and the latter only two species, placing the rest of older names as varieties. Specimen scarcity and lack of nomenclatural types seem to have been largely responsible for the nomenclatural instability in *Ceratozamia*. This issue was clarified by Stevenson and Sabato (1986).

Given the considerable increase in activities dedicated to the exploration of cycad diversity in Mexico (the target of the main taxonomic interest for the last 40 years) and the unstable taxonomy and nomenclature in *Ceratozamia*, we present a taxonomic clarification of the *Ceratozamia* species found along the Sierra Madre Oriental (SMO) from North Tamaulipas to North Oaxaca, which represent areas of endemism for this biogeographic province.

## Materials and methods

**Figure 1.**
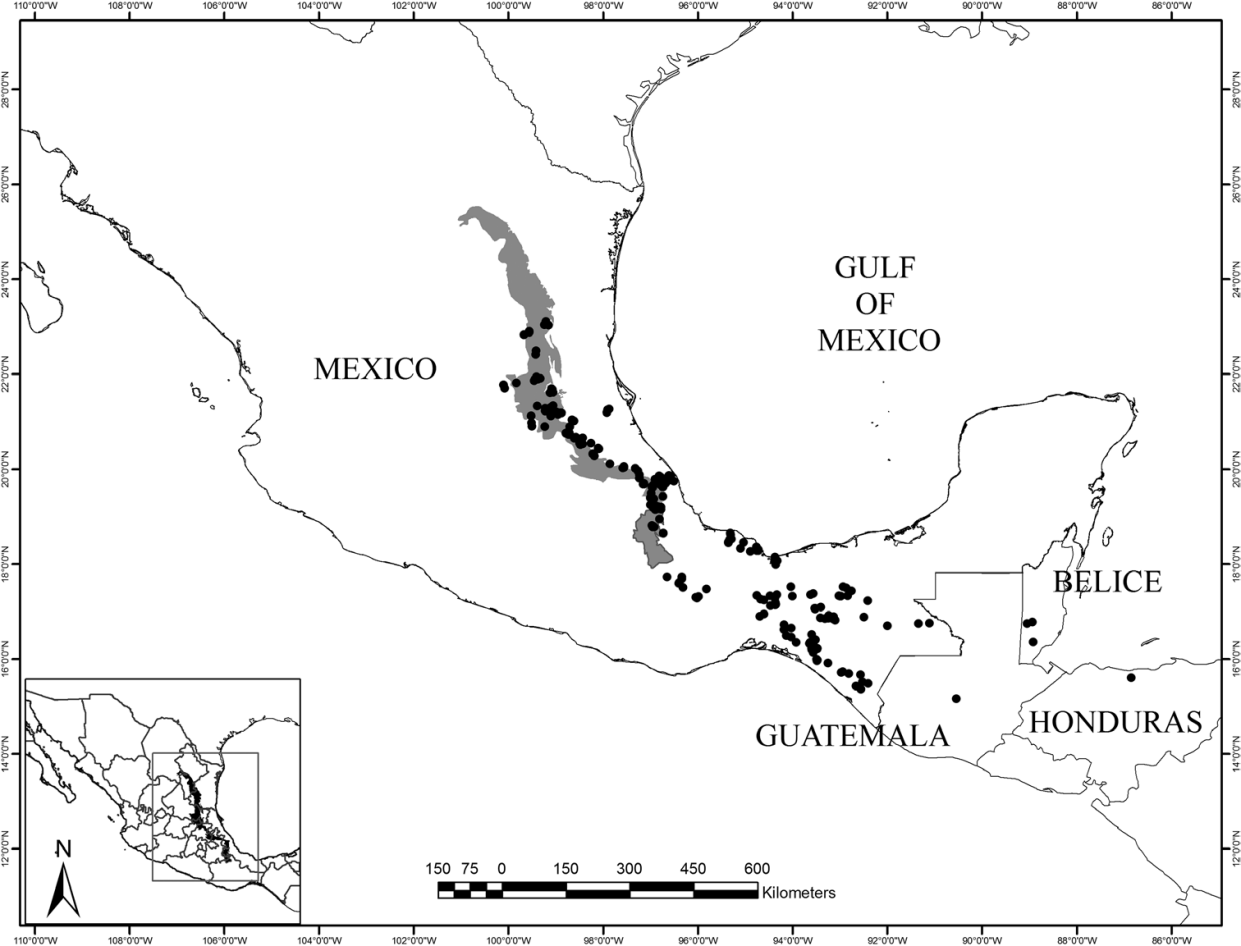
Map of the entire distribution of Ceratozamia in relation to the Sierra Madre Oriental (in grey).

Species-level circumscription is based on results of a phylogenetic analyses performed for *Ceratozamia* species that included quantitative and qualitative morphological data, molecular evidence, as a well as analyses of herbarium specimens (Martínez-Domínguez 2018). We present the taxonomic treatment for a part of the genus, corresponding to a well-delimited biogeographic region (Fig. 1). We examined herbarium specimens deposited in the following herbaria: CHAPA, CIB, ENCB, FCME, FTG, GH, HEM, IEB, K, LE, LSU, MB, MICH, MEXU, MO, NY, P, SERO, SLPM, U, UAT, US, XAL and XALU. This information was complemented by a review of the original taxonomic accounts for the relevant *Ceratozamia* species and additional data collected during fieldwork to evaluate diagnostic characters. The corresponding populations were monitored during four years (2014-2017) to evaluate reproductive structure characters, as well as some ontogenetic stages in these characters. In all cases, nomenclatural types were reviewed by all authors or at least one of them.

## Results

*Vegetative morphology*. Stems of the species here studied are epigeous and erect. They become decumbent with age and may develop two or more apices, with the exception of *Ceratozamia latifolia*, *C. hildae*, *C. kuesteriana* and *C. zaragozae*, which have semi-epigeous stems.

**Figure 2.**
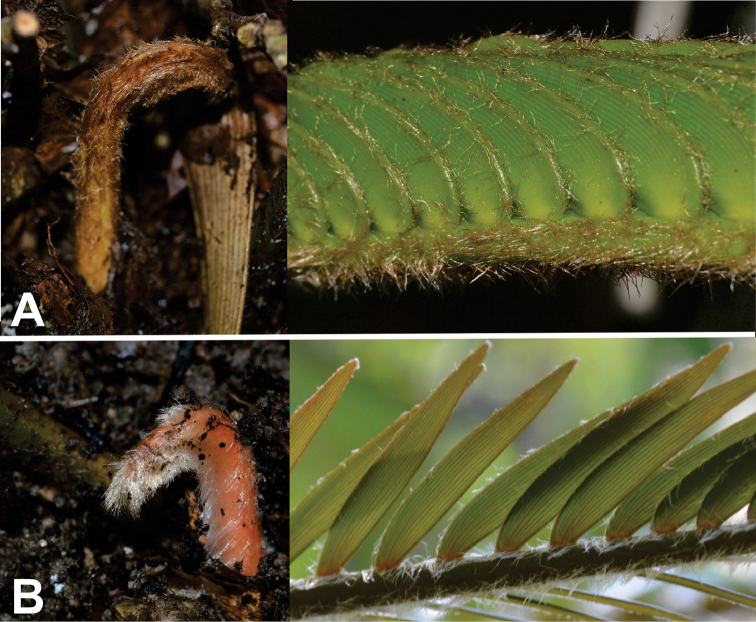
Trichome colour variation. **A** brown, *Ceratozamia tenuis* **B** white, *C. sabatoi*.

*Ceratozamia* species can produce 4 to 15 leaves per year and each apex can carry up to 100 leaves. *C. hildae* and *C. latifolia* are exceptional in this respect because they only produce 1 to 3 leaves per year. Leaves of most species reach up to 2.80 m long. The longest leaves are present in *C. mexicana*, *C. hildae* and *C. tenuis*, whereas the shortest can be observed in *C. latifolia* and *C. zaragozae*. New leaf colour can vary from light green to yellowish, as in *C. brevifrons*, to dark green (blackish) as in *C. tenuis* or reddish-brown as in *C. chamberlainii*. Leaf colour can be persistent in some species and remain at the leaf base, margins, nerves and/or undersides of leaflets and the leaf rachis and/or petiole as in *C. latifolia*, *C. chamberlainii*, *C. kuesteriana* and *C. fuscoviridis*. In other species, leaf colouration can disappear at maturity as in *C. totonacorum*. At emergence, the leaf petiole and rachis have abundant trichomes of two types: brown for species distributed in Central or South Sierra Madre Oriental with the exception of *C. decumbens* and white for species distributed in North SMO (Fig. 2).

In most species, presence of prickles in the petiole and rachis is highly variable intraspecifically. Prickles can be long as in *C. tenuis* where they are up to 0.6 cm long or very short as in *C. sabatoi* of approximately 0.1 cm. However, the shape of prickles is a constant character within populations. Two forms have been identified: 1) thin and easily detached and 2) robust, wide and hard to separate from the plant.

**Figure 3.**
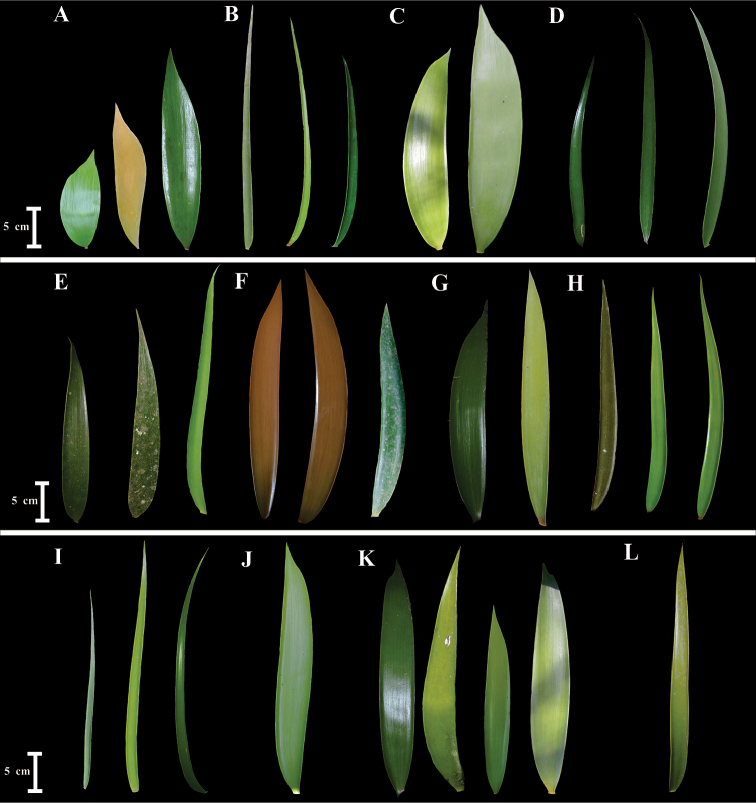
Shapes of leaflets. **A** *C. latifolia* **B** *C. kuesteriana* **C** *C. morettii* **D** *C. sabatoi* **E** *Ceratozamia brevifrons* **F** *C. chamberlainii* **G** *C. decumbens* **H** *C. fuscoviridis* **I** *C. tenuis* **J** *C. totonacorum* **K** *C. delucana* **L** *C. mexicana*.

**Figure 4.**
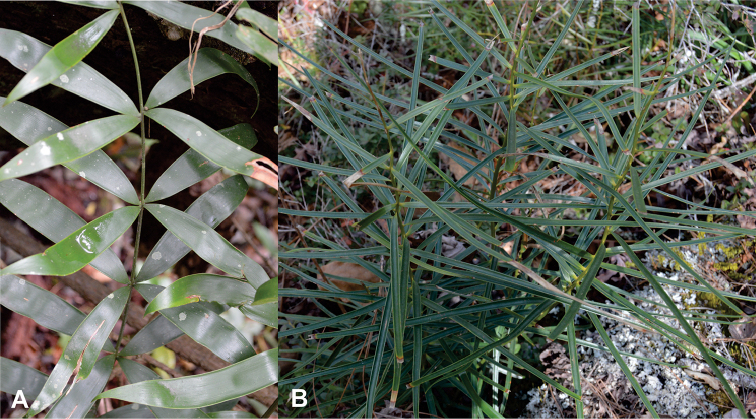
Types of leaves and leaflets. **A** Linear petiole and rachis with clustered leaflets **B** Twisted petioles and rachis, with opposite to subopposite leaflets.

Leaflet form can be lanceolate, oblong or linear. This character can be polymorphic within and between populations as in *Ceratozamia delucana* (Fig. 3). Leaflet width can vary from narrow, 0.4–0.7 cm in *C. zaragozae* (Fig. 4), to broad, up to 5 cm as in *C. latifolia* and *C. decumbens*. Other characters with taxonomic value are leaflet symmetry and texture. However, these characters can be polymorphic as in *C. delucana*. Leaflet texture is generally papyraceous as in *C. latifolia* or coriaceous as in *C. brevifrons*. In contrast, leaflets in *C. hildae* and *C. zaragozae* are membranaceous. Leaflet symmetry can be curved as in *C. fuscoviridis* or straight from the base to the apex as in *C. latifolia*. In terms of leaflet lamina shape, some species are abaxially curved, while others are planar, as *C. fuscoviridis* and *C. decumbens*, respectively.

*Reproductive morphology.* All qualitative characters of both the pollen strobili and the ovulate strobili are not very polymorphic within and between populations, with the exception of strobilar apices in ovulate plants for certain species, e.g. *Ceratozamia brevifrons*, which can have acuminate and apiculate apex. Several reproductive characters contribute to diagnosable characters separating closely related taxa.

**Figure 5.**
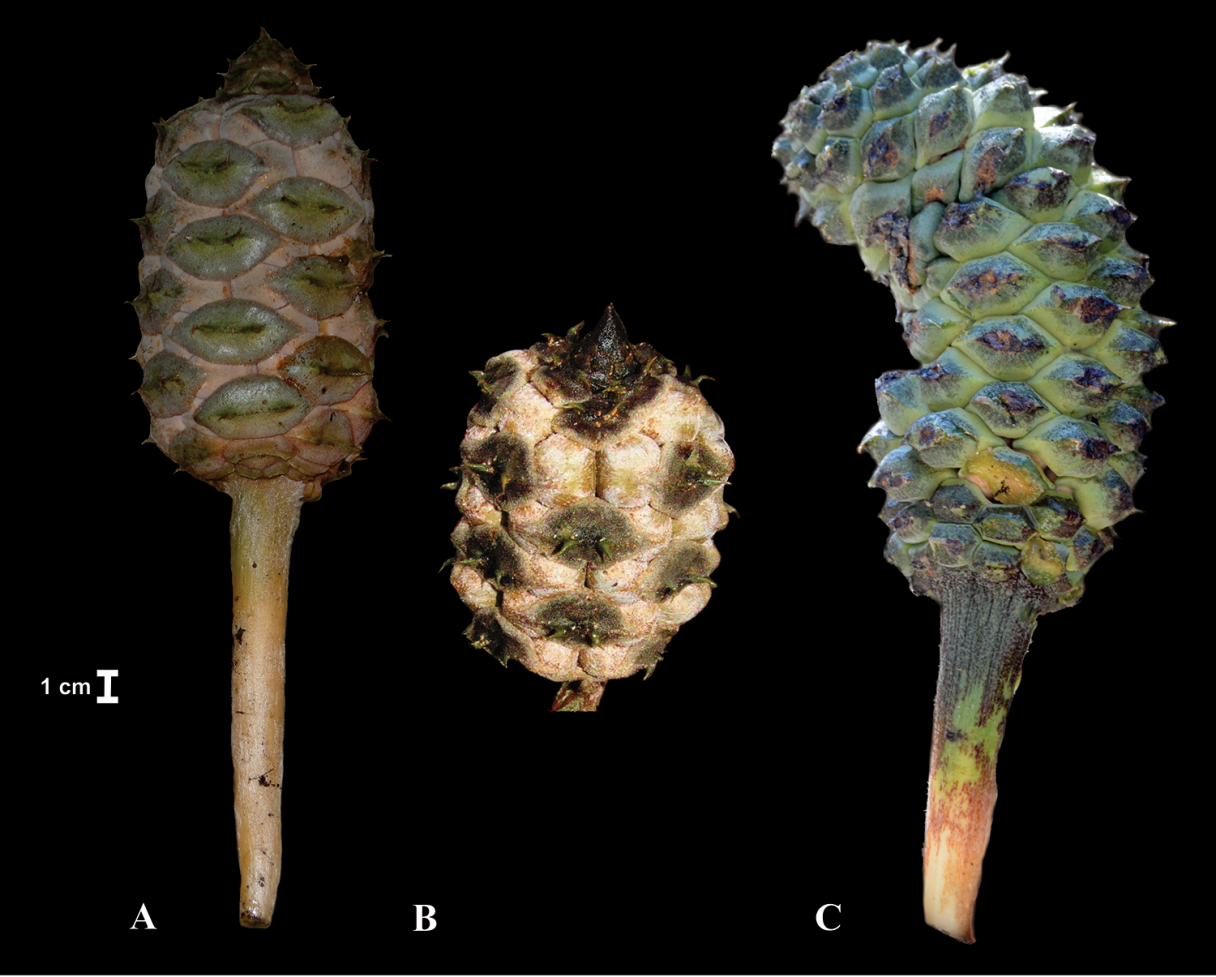
Shapes of ovuliferous strobili. **A** *Ceratozamia latifolia* **B** *C. decumbens* **C** *C. zaragozae*.

Ovuliferous strobili provide most of the diagnostic/differential in *Ceratozamia*. The most relevant of these characters are the colour of the strobilus, the shape of the fertile part of the entire strobilus, horn form in the distal end of sporophylls and colour of the sarcotesta (Fig. 5). The colour of ovuliferous strobili at maturity vary from dark green, as in *C. mexicana*, to wine red, as in *C. decumbens*. The shape of the strobilus is cylindric in most species, with the exception of *C. morettii* and *C. latifolia* where it is globose. In *C. delucana*, this character is polymorphic as both shapes can occur.

**Figure 6.**
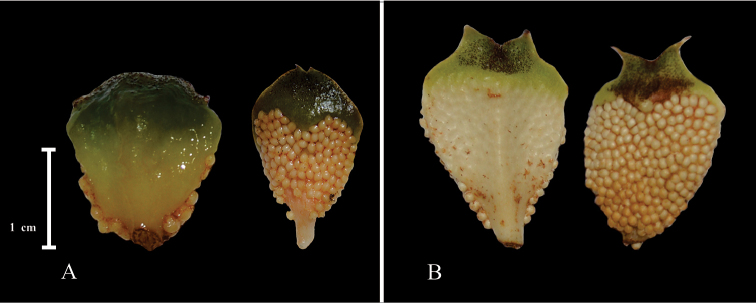
Microsporophylls. **A** *C. sabatoi* **B** *C. brevifrons*.

**Figure 7.**
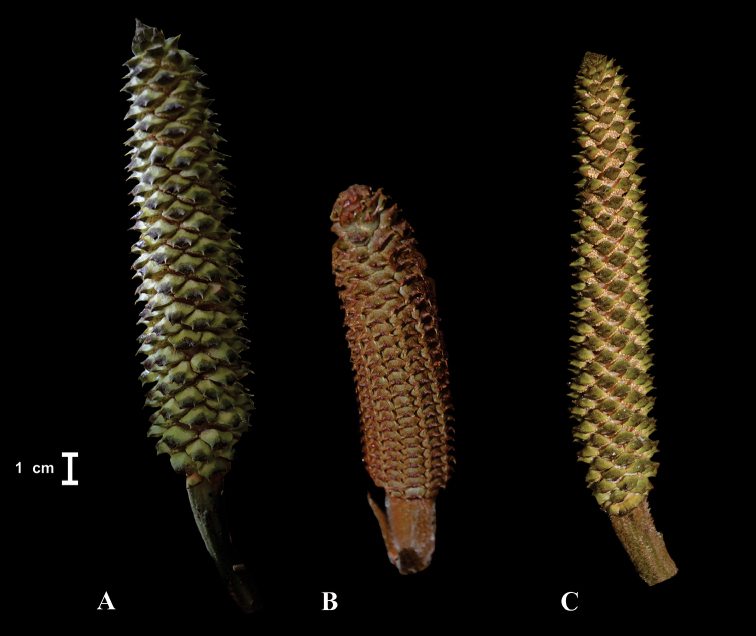
Shapes of polliniferous strobilus. **A** *Ceratozamia sabatoi* **B** *C. hildae* **C** *C. kuesteriana*.

Finally, polliniferous strobili provide two diagnostic characters. First, the distal end of sporophylls can be recurved upon itself or not curved (Fig. 6). Second, the colour may be greenish, yellowish with abundant brown or blackish trichomes or reddish-brown or yellowish-brown (Fig. 7). This character needs to be coded before the shedding of pollen. This is because mature pollen strobili turn yellow or cream-coloured and lose their characteristic colouration upon pollen shedding. The microsporangia cover the whole lower part of the sporophylls in such a crowded manner that they become angular, often irregular in shape and encroached upon one another. Therefore, the arrangements of microsporangia are not informative.

### Key to the species of *Ceratozamia* for the Sierra Madre Oriental

**Table:** 

1	Leaflets clustered	***C. hildae***
–	Leaflets evenly spaced, opposite to subopposite	**2**
2	Rachis and petiole twisted	***C. zaragozae***
–	Rachis and petiole straight	**3**
3	Leaflets linear to lanceolate, 0.4–2 cm wide	**4**
–	Leaflets lanceolate to oblong, >2 cm wide	**5**
4	Leaflet lamina caniculate	**6**
–	Leaflet lamina flat	**7**
5	Petiole with prickles, stem epigeous	**8**
–	Petiole unarmed, stem semi-epigeous	***C. latifolia***
6	Stem semi-hypogeous, new leaves reddish-brown, whitish pubescence, ovuliferous strobilus greyish-light green with black trichomes	***C. kuesteriana***
–	Stem epigeous, new leaves dark-green, brown pubescence, ovuliferous strobilus dark green with blackish trichomes	***C. tenuis***
7	Leaves descending, leaflets linear, microsporophyll with distal end recurved, ovuliferous strobilus blue green with blackish trichomes	***C. sabatoi***
–	Leaves ascending, leaflets lanceolate, microsporophyll with distal end straight, ovuliferous strobilus brown-green with dark brown trichomes at maturity	***C. fuscoviridis***
8	Leaves ascending	***C. delucana***
–	Leaves descending	**9**
9	Leaflets lanceolate	**10**
–	Leaflets oblong	**11**
10	Leaflets keeled, petiole with long (0.3–0.6 cm), prickles robust and abundant (>50), ovuliferous strobilus greenish-yellow with brown to blackish trichomes	***C. brevifrons***
–	Leaflets plane, petiole with short (0.1–0.2 cm), prickles thin and sparse (<40), ovuliferous strobilus dark green with black and grey trichomes	***C. mexicana***
11	New leaves light green, ovuliferous strobilus globose and green with blackish trichomes	***C. morettii***
–	New leaves brown to reddish-brown, ovuliferous strobilus cylindric and brown to wine red	**12**
12	New leaves light brown, petiole with long prickles	***C. totonacorum***
–	New leaves dark-brown to reddish brown, petiole with short prickles	**13**
13	Veins reddish-brown, ovuliferous strobilus light greyish-brown with abundant reddish-brown trichomes and acuminate apex	***C. chamberlainii***
–	Veins light green, ovuliferous strobili wine with blackish trichomes and acute apex	***C. decumbens***

### Taxonomic treatment

#### 1. Ceratozamia brevifrons

Taxon classificationPlantaeCycadalesZamiaceae

Miq. Tijdschr. wis-en natuurk Wet. 1: 41–42. 1847.

Figures 3E , 6B

##### Type.

MEXICO. Veracruz: Alto Lucero de Gutiérrez Barrios, Apr. 2005, *S. Avendaño R. 5699* (neotype, designated by Vovides et al. 2012, XAL)

##### Description.

*Stem* epigeous, erect, 20–70 cm in length, 15–40 cm in diameter. *Cataphylls* persistent, densely tomentose at emergence, reddish-brown and glabrous at maturity, triangular, apex acuminate, 2–5 × 1.5–4 cm at base. *Leaves* 6–36, descending, 58–173.5 cm, yellowish-green at emergence with a brown pubescence, glabrous at maturity. *Petiole* terete, straight, 20–56 cm, armed with short and robust prickles, green in adult leaves. *Rachis* terete, straight, 35–125.5 cm, armed with prickles, green in adult leaves. *Leaflets* 13–38, lanceolate, abaxially curved and planar, basally falcate, coriaceous, flat, opposite to subopposite, keeled, light green, adaxial and abaxial surfaces glabrous, acuminate apex, symmetric to asymmetric apex, attenuate at base, with conspicuous and light green veins; median leaflets 15.5–41 × 2–4.1 cm, 0.5–3.2 cm between leaflets; articulations yellow, 0.6–1.7 cm wide. *Polliniferous strobilus* solitary, cylindrical, erect, 22–31 cm in length, 5–7 cm in diameter, greenish-yellow at emergence, greenish-yellow with brown to blackish pubescence at maturity; peduncle tomentose, reddish-brown to light-brown, 5.5–9.5 cm in length, 1.8–2.2 cm in diameter; microsporophylls 1.6–2.3 × 1.1–1.5 cm, distal face not recurved. *Ovuliferous strobilus* solitary, cylindrical, erect, 26.5–30 cm in length, 9.8–12 cm in diameter, green with blackish pubescence at emergence, greenish-yellow with brown to blackish trichomes at maturity, acuminate and apiculate apex; peduncle tomentose, brown to reddish-brown, 6–12 cm in length, 2.1–2.4 cm in diameter; megasporophylls 80–154, 1.5–2.5 × 2.3–3.0 cm, prominent distal face, right angle between horns. *Seeds* ovoid, sarcotesta whitish-yellow to yellow when immature, light brown at maturity, 2.5–3 cm in length, 1.7–2 cm in diameter.

##### Distribution and habitat.

**Figure 8.**
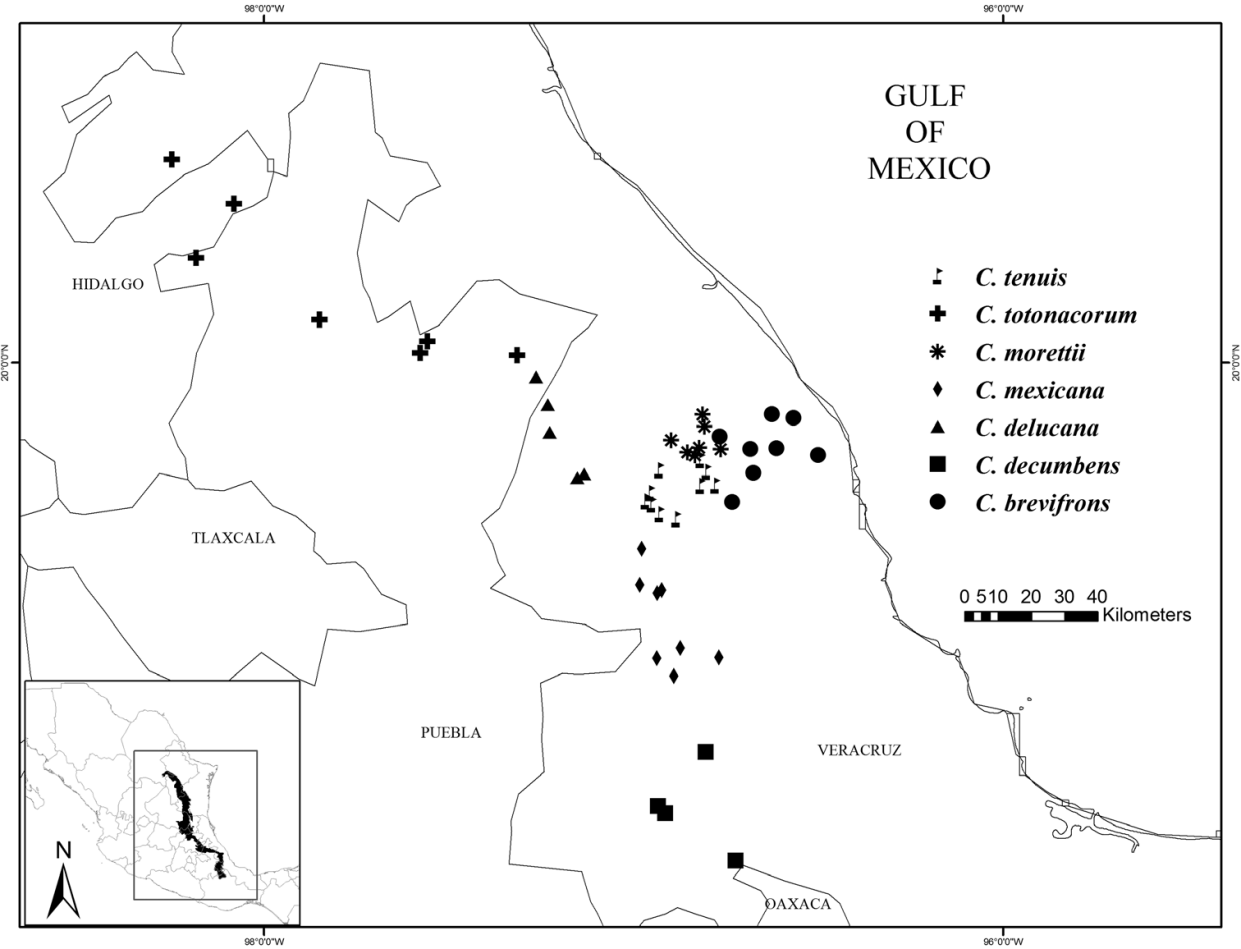
Distribution map of *Ceratozamia tenuis*, *C. totonacorum*, *C. morettii*, *C. mexicana*, *C. delucana*, *C. decumbens* and *C. brevifrons*.

Endemic to Mexico and only known from the State of Veracruz, in the vicinity of the Sierra de Chiconquiaco, at the transition zone between cloud forest and oak forest, between 500 and 1,350 m of elevation (Fig. 8).

##### Etymology.

The epithet is derived from referring to its relatively short leaves.

##### Distinguishing features.

This species is easily distinguished from its congeners by having leaflets adaxially keeled, falcate, basally falcate and coriaceous, petioles armed with short, robust prickles, ovulate strobili greenish-yellow with brown to blackish trichomes at maturity and megasporophylls with a prominent distal face and right angle between horns.

##### Specimens examined.

MEXICO. **Veracruz**: Alto Lucero de Gutiérrez Barrios, *A. P. Vovides 119* (XAL), *D. Jimeno-Sevilla 694* (XAL), *F. Nicolalde-Morejón & L. Martínez-Domínguez 2027-2046* (CIB), *G. Castillo-Campos 1297* (XAL), *J. Rees 1636* (MO, XAL), *1641, 1642, 1675* (XAL), *6345* (IEB), *L. Martínez-Domíguez & F. Nicolalde-Morejón 130-133, 216, 229-309* (CIB), *M. Vázquez-Torres 4790* (CIB), *T. W. Walters 2001-02-A, B* (XAL); Chiconquiaco, *F. Nicolalde-Morejón & L. Martínez-Domínguez 2237-2241* (CIB), *L. Martínez-Domíguez & F. Nicolalde-Morejón 556-560* (CIB); Juchique de Ferrer, *A. P Vovides 682* (XAL), *G. Castillo-Campos 1710, 1763, 1768, 1815, 1824, 1981* (XAL), *M. Vázquez-Torres 8633* (CIB); Vega de Alatorre, *B. Guerrero & J. I. Calzada 1826* (XAL), *G. Castillo-Campos 2033* (XAL).

##### Taxonomic comments.

This species name implies a long history of synonymy, which describes a series of substantial taxonomic disagreements. In the pioneer taxonomic treatments for the genus, *C. brevifrons* was considered as a synonym to *C. mexicana* (Miquel 1861, 1868-1869; Regel 1876a; Thiselton-Dyer 1884; Schuster 1932), whereas in the most recent treatment it was placed as a synonym of C. mexicana var. mexicana (Vovides et al. 1983). In 2012, Vovides and collaborators removed the binomial from this synonymy after finding plants in the wild, which morphologically corresponded to its original description.

#### 2. Ceratozamia chamberlainii

Taxon classificationPlantaeCycadalesZamiaceae

Mart.-Domínguez, Nic-Mor. & D.W. Stev. Phytotaxa 317(1): 17–28. 2017.

Figure 3F

##### Type.

MEXICO. San Luis Potosí: Xilitla, 20 Mar. 2016, *L. Martínez-Domínguez et al. 933* ♀ (holotype: CIB; isotypes: MEXU, NY).

##### Description.

*Stem* epigeous, erect and decumbent, 20–60 cm in length, 15–30 cm in diameter. *Cataphylls* persistent, densely tomentose at emergence, reddish-brown and glabrous at maturity, triangular, apex acuminate, 2.5–5.5 × 1.5–3.5 cm at base. *Leaves* 5–40, descending, 100–207 cm, reddish-brown at emergence with whitish-grey pubescence, glabrous at maturity. *Petiole* terete, straight, 30–69 cm, armed with short and robust prickles, blackish-brown in adult leaves. *Rachis* terete, straight, 65–144 cm, armed with prickles, reddish-brown in mature leaves. *Leaflets* 20–42, oblong, planar and abaxially curved, not basally falcate, coriaceous, flat, opposite to subopposite, plane, dark green, adaxial and abaxial surfaces glabrous, acuminate and asymmetric apex, attenuate at base, with conspicuous and reddish-brown veins; median leaflets 20–37 × 2.3–4 cm, 1.6–3.5 cm between leaflets; articulations generally reddish-brown, 0.5–1.3 cm wide. *Polliniferous strobilus* generally solitary (1–2), cylindrical, erect, 26–31 cm in length, 4.8–6 cm in diameter, greenish-brown at emergence, greenish with reddish-brown pubescence at maturity; peduncle tomentose, reddish-brown, 6–10.3 cm in length, 1.6–2 cm in diameter; microsporophylls 1.6–2.3 × 1.1–1.5 cm, not recurved distal face. *Ovuliferous strobilus* cylindrical, erect, 28–30 cm in length, 9–10.5 cm in diameter, greyish-green with reddish-brown pubescence at emergence, light greyish-brown with reddish-brown trichomes at maturity, acuminate apex; peduncle tomentose, brown to reddish-brown, 4.5–11 cm in length, 1.3–2.5 cm in diameter; megasporophylls 72–190, 1.5–2.5 × 2.3–3.0 cm, prominent distal face, acute angle between horns. *Seeds* ovoid, sarcotesta whitish-red to pink when immature, light brown at maturity, 3.0–4.0 cm in length, 0.6–1.2 cm in diameter.

##### Distribution and habitat.

**Figure 9.**
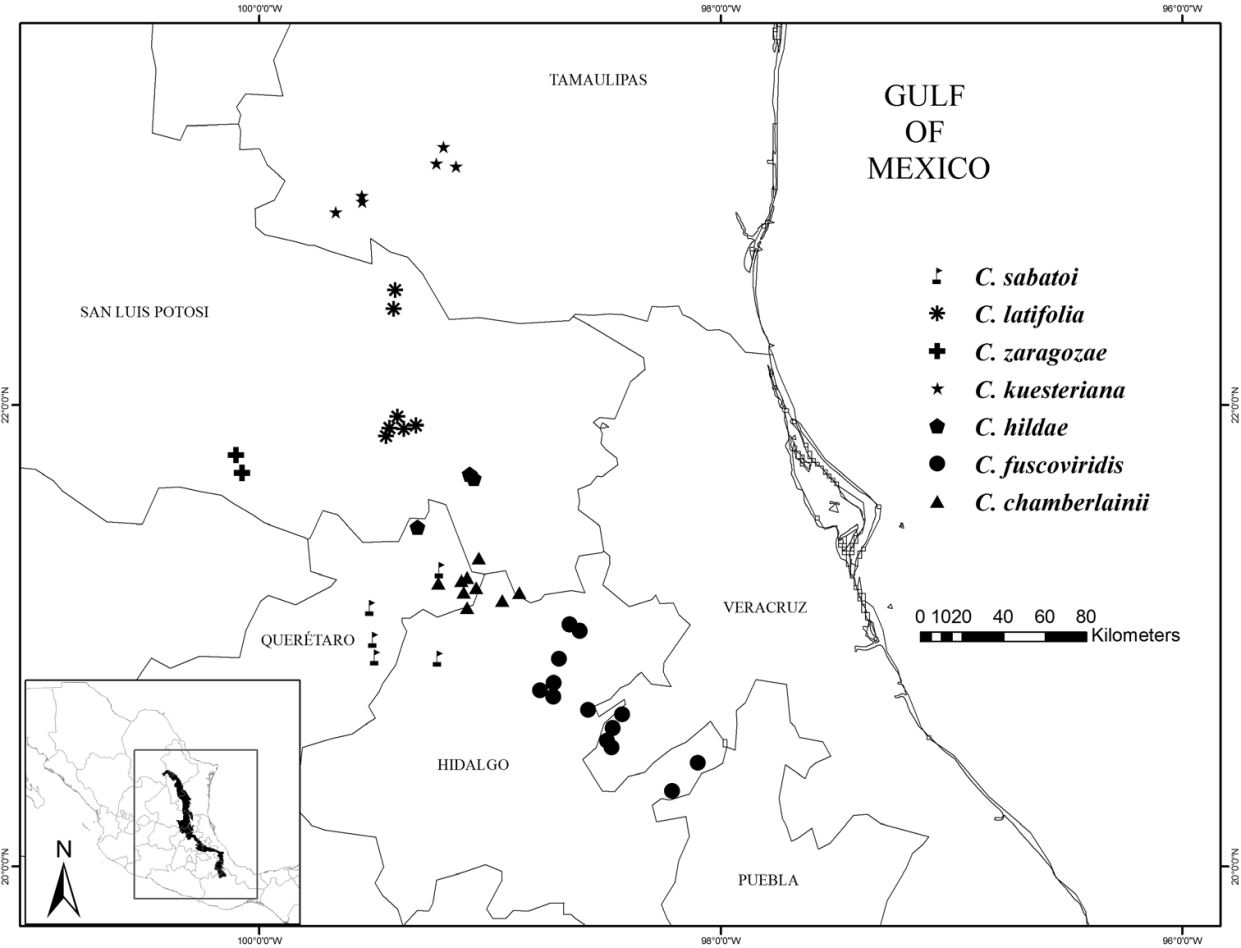
Distribution map of *Ceratozamia sabatoi*, *C. latifolia*, *C. zaragozae*, *C. kuesteriana*, *C. hildae*, *C. fuscoviridis* and *C. chamberlainii*.

Its range includes the states of San Luis Potosí, Querétaro and Hidalgo (Fig. 9). *Ceratozamia chamberlainii* occurs in the cloud forest on rocky outcrops at 900–1,200 m.

##### Etymology.

The specific epithet honours Charles Joseph Chamberlain in recognition of his outstanding contributions to knowledge of the biology of the cycads and his fieldwork on Mexican cycads.

##### Distinguishing features.

This species is distinguished by its petioles and rachis with abundant, short prickles; reddish-brown leaves at emergence with coloured articulations at the leaflet attachment to the rachis in adult leaves. Leaflets are oblong and coriaceous with conspicuous, reddish-brown veins.

##### Specimens examined.

MEXICO. **Hidalgo**: Chapulhuacán, *Alcántara-Ayala 3650* (FCME), *F. Nicolalde-Morejón et al. 2200-2203* (CIB), *L. Martínez-Domínguez et al. 429*–*430* (CIB), *441–443* ♀ (CIB), *447* ♂ (CIB), *Vite-Reyes et al. 23* (XAL); La Misión, *Castro-Castro et al*. *1017* (XAL), *Vite-Reyes et al. 20* (XAL). **Querétaro**: Landa de Matamoros, *A. P. Vovides 1291* (XAL, MEXU), *1288, 1290*, 2000 (XAL), *E. Carranza 633* (IEB), *3119* (MEXU), *F. Nicolalde-Morejón et al. 2192-2199* (CIB); *L. Martínez-Domínguez et al. 379, 405* ♂ (CIB), *380–404* (CIB); *L. Martínez-Domínguez et al. 408–426* (CIB), *427, 428* ♂ (CIB), *Rubio Hiram 6332*, *6334* (IEB), *647* (XAL). **San Luis Potosí**: Tamazunchale, *C. L. Lundell 7235* (IEB); Xilitla, *F. Nicolalde-Morejón et al. 2407-2420* (CIB), *L. Martínez-Domínguez et al. 924*–*932* (CIB); *T. W. Walters, TW-2001-04-A* (XAL).

#### 3. Ceratozamia decumbens

Taxon classificationPlantaeCycadalesZamiaceae

Vovides, Avendaño, Pérez-Farr. & Gonz.-Astorga. Novon 18 (1): 109–114. 2008.

Figure 3G

##### Type.

MEXICO. Veracruz: Naranjal, 8 Apr. 2005, *S. Avendaño & G. Alducin 5706* (holotype: XAL; isotypes: HEM, MO).

##### Description.

*Stem* epigeous, erect and decumbent, 10–40 cm in length, 10–25 cm in diameter. *Cataphylls* persistent, densely tomentose at emergence, reddish-brown and partially tomentose at maturity, triangular, apex acuminate, 1.5–3 × 2–4.2 cm at base. *Leaves* 2–7, descending, 80–190 cm, reddish-brown at emergence, whitish-grey pubescence, glabrous at maturity. *Petiole* terete, straight, 40–100 cm, armed with short and thin prickles, greenish-brown in adult leaves. *Rachis* terete, straight, 40–123 cm, armed with prickles and occasionally unarmed, brown and green in adult leaves. *Leaflets* 8–24, oblong, mostly planar, not basally falcate, coriaceous, flat, opposite to subopposite, plane, green, adaxial and abaxial side glabrous, acuminate apex, symmetric and asymmetric apex, attenuate at base, with conspicuous and light green veins; median leaflets 23–47.5 × 2.8–5 cm, 2.6–6.5 cm between leaflets; articulations brown, 0.7–1.2 cm wide. *Polliniferous strobilus* solitary, cylindrical, erect, 20–23 cm in length, 4–4.5 cm in diameter, greenish-yellow at emergence, greenish with reddish-brown pubescence at maturity; peduncle tomentose, reddish-brown to brown, 8–8.5 cm in length, 1.2–1.5 cm in diameter; microsporophylls 1–1.9 × 1–1.4 cm wide, non-recurved distal face. *Ovuliferous strobilus* solitary, cylindrical, erect, 9–11 cm in length, 7–8 cm in diameter, wine at emergence, wine with blackish trichomes at maturity, acute apex; peduncle tomentose, brown, 3–4 cm in length, 1–1.2 cm in diameter; megasporophylls 24–49, 2.3–2.5× 2–3 cm, truncate distal face, right angle between horns. *Seeds* ovoid, sarcotesta whitish-red when immature, light brown at maturity, 1.2–2 cm, 1.2–1.5 cm in diameter.

##### Distribution and habitat.

Endemic to a small mountain range in central Veracruz, 450–1,000 m elevation (Fig. 8). The vegetation type of the habitat is mountain tropical forest and cloud forest on karstic rocks.

##### Etymology.

The epithet alludes to the decumbent nature of trunks in older mature plants.

##### Distinguishing features.

*Ceratozamia decumbens* is distinguished by its oblong coriaceous, mostly planar and basally falcate leaflets; ovulate strobilus wine red with blackish trichomes at maturity and acute apex.

##### Specimens examined.

MEXICO. **Veracruz**: Atoyac, *R. Acevedo R. 728* (XAL); Coetzala, *A. Rincón G. 2798* (MEXU, XAL), *L. Martínez-Domínguez et al. 655-683* (CIB); Ixtaczoquitlán, *A. Pérez P. 282* (XAL); Naranjal, *A. P. Vovides 751* (XAL), *Brigada T. Walters s/n* (XAL), *J. Rees 1690* (XAL), *S. Avendaño R. & G. Alducin 5706* (XAL), *T. W. Walters 41277, 41308, 41397* (XAL); Tequila, *Jaime E. Rivera Hdez. & Antoeván Vergara V. 4195* (MEXU, XAL), *F. Nicolalde-Morejón et al. 2259, 2260* (CIB), *L. Martínez-Domínguez et al. 684-703*; Tezonapa, *M. A. García B. 980* (XAL), *R. Robles G. 882* (XAL).

#### 4. Ceratozamia delucana

Taxon classificationPlantaeCycadalesZamiaceae

Vázq.-Torres, Moretti & Carvajal-Hernández. Delpinoa, 50–51, 129–133. 2013.

Figure 3K

##### Type.

MEXICO. Veracruz: Atzalan, 20 Jan. 2012, *M. Vázquez-Torres & C. Carvajal-Hernández 10200* (holotype: CIB; isotypes: XAL, XALU).

##### Description.

*Stem* epigeous, erect and decumbent, 20–90 cm in length, 25–40 cm in diameter. *Cataphylls* persistent, densely tomentose at emergence, reddish-brown and partially tomentose at maturity, triangular, apex acuminate, 2–5.5 × 2.5–4.5 cm at base. *Leaves* 10–100, ascending, 106–223 cm, yellowish-green at emergence, brown pubescence, glabrous at maturity. *Petiole* terete, straight, 30–87 cm, armed with short and thin prickles, green in adult leaves. *Rachis* terete, straight, 60–150 cm, armed with prickles, green in adult leaves. *Leaflets* 20–43, lanceolate and oblong, planar and abaxially curved, basally falcate to non-basally falcate, papyraceous to coriaceous, flat, opposite to subopposite, plane, green, adaxial side glaucous and glabrous and abaxial side glaucous, acuminate apex, symmetric to asymmetric apex, attenuate at base, with conspicuous and light green veins; median leaflets 22–45 × 2.3–4.6 cm, 1.5–5 cm between leaflets; articulations green, 0.6–1.6 cm wide. *Polliniferous strobilus* solitary, cylindrical, erect, 24–31 cm in length, 5.5–7.6 cm in diameter, greenish-yellow at emergence, greenish-yellow with blackish pubescence at maturity; peduncle tomentose, reddish-brown to light-brown, 3.5–12.5 cm in length, 1.3–2 cm in diameter; microsporophylls 1.5–2.5 × 1.3–2 cm, non-recurved distal face. *Ovuliferous strobilus* solitary, cylindrical and globose, erect, 18–40 cm in length, 10–12.5 cm in diameter, dark green with blackish pubescence at emergence, green generally glabrous at maturity, acute apex; peduncle tomentose, brown to reddish-brown, 5.2–15 cm in length, 1.8–2.2 cm in diameter; megasporophylls 72–182, 2.5–4.2 × 2.3–3.5 cm, truncate distal face, right angle between horns. *Seeds* ovoid, sarcotesta whitish-yellow to yellow when immature, light brown at maturity, 2.1–3 cm in length, 1.6–2.1 cm in diameter.

##### Distribution and habitat.

This species is known from the states of Veracruz and Puebla at 200–700 m in evergreen tropical forest (Fig. 8).

##### Etymology.

The epithet is in honour of Dr. Paolo De Luca, Professor at University of Naples Federico II and researcher in the biology of Mexican cycads.

##### Distinguishing features.

*Ceratozamia delucana* is highly variable and shares a number of characteristics with *C. morettii*. However, there are clear differences in their ovulate strobili. In *C. delucana*, ovulate strobili are green and generally without trichomes at maturity and have an acute apex, whereas in *C. morettii* they are green with blackish trichomes at maturity and have an apiculate apex. Additionally, *C. delucana* is larger than *C. morettii*, with *C. delucana* having leaves up to 223 cm with up to 43 pairs of leaflets and ovulate strobili 18–40 cm long.

##### Specimens examined.

MEXICO. **Puebla**: Xochitlán de Vicente Suárez, *G. Villalobos & E. Guerrero C*. *325* (MEXU), *L. Martínez-Domínguez & F. Nicolalde-Morejón 587-616* (CIB). **Veracruz**: Atzalan, *F. Nicolalde-Morejón & L. Martínez-Domínguez 2125-2144* (CIB), *L. Martínez-Domínguez et al. 228-248* (CIB); Las Minas, *A. P. Vovides 427* (XAL), *C. Durán E. 6343* (IEB), *659* (MEXU, XAL), *C. Durán, P. Burgos, A. P. Vovides 658* (XAL), *660* (MEXU, XAL), *F. Nicolalde-Morejón & L. Martínez-Domínguez 2107-2124* (CIB), *L. Martínez-Domínguez et al. 168* (CIB), 249-260; Tlapacoyan, *Nevling & A. Gómez-Pompa 1083* (MEXU).

#### 5. Ceratozamia fuscoviridis

Taxon classificationPlantaeCycadalesZamiaceae

W. Bull. Retail List 154: 4. 1879.

Figure 3H

##### Type.

Hort. Bot. Glasnevin, 21 Mar. 1878 (accessioned 1881), *D. Moore s.n.* (neotype, designated by Calonje and Sennikov 2017, K). **Epitype** (designated here). MEXICO. Hidalgo: Molango, 31 Mar. 2015, *L. Martínez-Domínguez et al. 493* [♀strob.] (CIB). **Isoepitype** (designated here). *L. Martínez-Domínguez et al. 493* (MEXU).

Ceratozamia mexicana var. longifolia f. fuscoviridis (W. Bull) Schuster. Pflanzenr 99:132. 1932. Type: Based on *Ceratozamia fuscoviridis* W. Bull.

##### Description.

*Stem* epigeous, erect and decumbent, 20–90 cm in length, 25–40 cm in diameter. *Cataphylls* persistent, densely tomentose at emergence, reddish-brown and partially tomentose at maturity, triangular, apex acuminate, 2–4.5 × 2–3.5 cm at base. *Leaves* 10–70, ascending, 92–215 cm, light green and dark brown at emergence, whitish- grey pubescence, glabrous at maturity. *Petiole* terete, straight, 40–95 cm, armed with long and thin prickles, dark green in adult leaves. *Rachis* terete, straight, 65–150 cm, armed with prickles, green in adult leaves. *Leaflets* 28–67, lanceolate, abaxially curved, basally falcate, papyraceous, flat, opposite to subopposite, plane, green, adaxial and abaxial side glabrous, acuminate and symmetric apex, attenuate at base, with conspicuous, and light green and brown veins; median leaflets 16.6–42 × 1.3–2.1 cm, 0.6–2 cm between leaflets; articulations green and brown, 0.6–1.3 cm wide. *Polliniferous strobilus* solitary, cylindrical, erect, 26.5–28 cm in length, 5–8 cm in diameter, brownish-yellow at emergence, greenish-brown with reddish-brown pubescence at maturity; peduncle tomentose, reddish-brown to brown, 5–14.5 cm in length, 1.6–2.3 cm in diameter; microsporophylls 1.9–2.3 × 1.3–1.5 cm, non-recurved distal face. *Ovuliferous strobilus* solitary, cylindrical, erect or pendulous, 24–35 cm in length, 8.5–10.5 cm in diameter, green with brown pubescence at emergence, brown-green with dark brown trichomes at maturity, acuminate apex; peduncle tomentose, brown to reddish-brown, 4–10 cm in length, 1.8–2.2 cm in diameter; megasporophylls 99–143, 2.5–3.8× 2.3–3.3 cm, truncate distal face, obtuse angle between horns. *Seeds* ovoid, sarcotesta whitish-yellow to yellow when immature, light brown at maturity, 2–2.6 cm in length, 1.5–2 cm in diameter.

##### Distribution and habitat.

Endemic of south-central Carso Huasteco, from central Hidalgo mountain range, to southeast of Hidalgo, including the western portion of Veracruz at an elevation ranging between 1,800–1,900 m in cloud forest (Fig. 9).

##### Etymology.

The epithet alludes to the dark-brown colour of the leaf at emergence.

##### Distinguishing features.

This species is distinguished by lanceolate and papyraceous leaflets; petiole armed with long, thin prickles; ovulate strobilus brown-green with dark trichomes at maturity. This species is polymorphic within populations as it has light green or dark-brown leaf colour at emergence in all populations.

##### Specimens examined.

MEXICO. **Hidalgo**: Eloxochitlán, *I. Luna Vega 54716* (FCME), *O. Alcántara Ayala 54805* (FCME); Metztitlán, *J. L. López-García 449* (MEXU); Molango de Escamilla, *A. Vite-Reyes et al. 6* (XAL), *A. P. Vovides 1298, 1301* (XAL), *F. Nicolalde-Morejón et al. 2209-2211* (CIB), *L. Martínez-Domínguez et al. 485-514* (CIB), *R. Mayorga-Saucedo & O. Alcántara-Ayala s/n* (FCME), *T. W. Walters 2001-03-A* (XAL); Tenango de Doria, *I. Luna Vega 794* (FCME); Tlanchinol, *I. Luna Vega s/n* (FCME), *625, 789* (XAL), *F. Nicolalde-Morejón et al. 2204-2208* (CIB), *L. Martínez-Domínguez et al. 455-484* (CIB); Zacualtipán de Ángeles, *J. Rees 389* (FCME, MEXU), *1611* (XAL), *6339* (IEB). **Veracruz**: Huayacocotla, *D. Saavedra Millán 64* (FCME), *J. Palma G. 63* (XAL), *F. Nicolalde-Morejón et al. 2212-2214* (CIB), *L. Ballesteros & F. Ballesteros 460* (XAL), *L. G. Juárez G. 47* (XAL), *L. Martínez-Domínguez et al. 515-544* (CIB), *R. Hernández M. 1507* (MEXU, XAL), *V. Sosa 59* (XAL).

##### Taxonomic comments.

This binomial has experienced different reassignments and transferences since its informal publication as “*Ceratozamia fusca-viridis*” by Moore in 1878. This author considered it as a provisional name (“proviso nomen”) and thus was not validly published. The changes that this name has undergone are the following: synonymy under Ceratozamia mexicana var. longifolia (Thiselton-Dyer 1884); transference from variety to form (Schuster 1932); and attempted validation of the name in accordance with current nomenclatural rules (Osborne et al. 2006). Recently, though, Calonje and Sennikov (2017) have attributed authorship of the binomial to Bull (1879) who presented a brief description of the taxon in a commercial catalogue of plants.

#### 6. Ceratozamia hildae

Taxon classificationPlantaeCycadalesZamiaceae

G. P. Landry & M. C. Wilson. Brittonia 31(3): 422–424. 1979.

Figures 4A , 7B

##### Type.

Louisiana, Baton Rouge, cultivated at 5988 South Pollard Parkway (originally from several km N of Xilitla, San Luis Potosí, Mexico), *Landry G 76521* (holotype: GH; isotypes: FTG, LSU, MEXU, MICH, NY, US).

##### Description.

*Stem* semi-epigeous, erect, 10–20 cm in length, 10–15 cm in diameter. *Cataphylls* persistent, densely tomentose at emergence, reddish-brown and partially tomentose at maturity, triangular, apex acuminate, 2.1–3 × 0.8–1.5 cm at base. *Leaves* 2–7, ascending, 95–202 cm, reddish-brown at emergence with whitish-grey pubescence, glabrous at maturity. *Petiole* terete, straight, 43–89 cm, armed with thin and short prickles, greenish-brown and green in adult leaves. *Rachis* terete, straight, 60–130 cm, armed with prickles and occasionally unarmed, greenish-brown in adult leaves. *Leaflets* with 5–11 fascicles, 16–56 leaflets in total, oblong, mostly planar, basally falcate to non-basally falcate, membranaceous, flat, clustered, plane, green, adaxial and abaxial side glaucous, acuminate apex, symmetric and asymmetric apex, attenuate at base, with conspicuous and light green veins; median leaflets 14.5–24 × 2.4–5 cm, 6–15 cm between leaflets; articulations brown and green, 0.2–0.5 cm wide. *Polliniferous strobilus* solitary, cylindrical, erect, 8–10 cm in length, 2–2.5 cm in diameter, brown with reddish-brown pubescence at emergence, reddish-brown at maturity; peduncle tomentose, reddish-brown to brown, 6–7.5 cm in length, 0.9–1 cm in diameter; microsporophylls 0.8–1.2 × 0.6–0.9 cm, non-recurved distal face. *Ovuliferous strobilus* solitary, cylindrical, erect, 10–14 cm in length, 6–9 cm in diameter, green at emergence with brown trichomes, green with brown to blackish trichomes at maturity, acuminate apex; peduncle tomentose, brown, 5–6 cm in length, 1.2–1.5 cm in diameter; megasporophylls 36–77, 2–3.7 × 2–4 cm, prominent distal face, right angle between horns. *Seeds* ovoid, sarcotesta whitish-red when immature, light brown at maturity, 1.3–2.3 cm in length, 1.2–1.5 cm in diameter.

##### Distribution and habitat.

Endemic to Mexico in San Luis Potosí (Fig. 9), in the evergreen tropical forests.

##### Etymology.

The epithet is in honour of Hilda Guerra Walker, daughter of the original collector.

##### Distinguishing features.

*Ceratozamia hildae* differs from its congeners by its clustered leaflets. Besides, leaflets are membranaceous and oblong.

##### Specimens examined.

MEXICO. **Querétaro**: Jalpan de Serra, *A. P. Vovides 337* (XAL), *A. P. Vovides s/n* (IEB), *B. Servin 870*, *986* (MEXU), *6328, 6330* (IEB), *J. Rees s/n* (IEB), *312* (XAL). **San Luis Potosí**: Aquismón, *A. P. Vovides & J. Rees 312* (MEXU, XAL), *F. Nicolalde-Morejón et al. 2391-2406* (CIB), *L. Martínez-Domínguez et al. 910-923* (CIB), *S. Longoria s/n* (XAL), *T. W. Walters TW-2001-14-A* (MEXU, XAL), *TW-2001-14-B* (XAL), *TW-2001-14-C* (MEXU).

#### 7. Ceratozamia kuesteriana

Taxon classificationPlantaeCycadalesZamiaceae

Regel. Bull. Soc. Imp. Naturalistes Moscou 30: 187–188, t. 3. 1857.

Figures 3B , 7C

##### Type.

ex Horto Petropolitano, 1856, *Regel s.n.* (holotype, LE; isotypes: U).

##### Description.

*Stem* semihypogeous, erect, 10–30 cm in length, 10–25 cm in diameter. *Cataphylls* persistent, densely tomentose at emergence, reddish-brown and partially tomentose at maturity, triangular, apex acuminate, 1.5–4 × 2–3.5 cm at base. *Leaves* 1–11, ascending, 80–133 cm, reddish-brown at emergence, whitish-grey pubescence, glabrous at maturity. *Petiole* terete, straight, 30–72 cm, armed with thin and short prickles, greenish-brown in adult leaves. *Rachis* terete, straight, 40–72 cm, armed with prickles and rarely unarmed, greenish-brown in adult leaves. *Leaflets* 22–50, linear, adaxially curved, basally falcate, papyraceous, caniculate, opposite to subopposite, plane, green, adaxial and abaxial side glabrous, acuminate apex, symmetric apex, attenuate at base, with conspicuous and light green veins; median leaflets 17–32 × 0.6–1 cm 0.3–2.5 cm between leaflets; articulations brown, 0.2–0.8 cm wide. *Polliniferous strobilus* solitary, cylindrical, erect, 11–15 cm in length, 2.2–3 cm in diameter, greenish-yellow with brown pubescence at emergence, yellowish-brown with reddish-brown pubescence at maturity; peduncle tomentose, reddish-brown to brown, 9–14 cm in length, 0.9–1 cm in diameter; microsporophylls 0.6–1.1 × 0.6–1 cm, not recurved distal face. *Ovuliferous strobilus* solitary, cylindrical, erect, 13–21 cm in length, 7–9 cm in diameter, green at emergence with brown trichomes, greyish-light green with black trichomes at maturity, acuminate apex; peduncle tomentose, brown, 11–15 cm in length, 1–2 cm in diameter; megasporophylls 52–90, 2.5–4 × 2.3–3.5 cm, truncate distal face, obtuse angle between horns. *Seeds* ovoid, sarcotesta whitish-red when immature, light brown at maturity, 1.5–2.2 cm in length, 1.2–1.8 cm in diameter.

##### Distribution and habitat.

Endemic to Mexico in Tamaulipas at 1,100–1,500 m (Fig. 9), in pine-oak and cloud forests.

##### Etymology.

The specific epithet is in honour of Baron K. von Kuester, 19^th^ century plant collector.

##### Distinguishing features.

This species is distinguished by leaflets linear, planar and abaxially curved, not basally falcate, papyraceous, flat and symmetric apex.

##### Specimens examined.

MEXICO. **Tamaulipas**: Gómez Farías, *A. Gómez-Pompa 2029* (MEXU), *A. P. Vovides 771, 772, 791, 800, 801* (XAL), *F. González-Medrano et al. 3362* (MEXU, MO), *F. González-Medrano & E. Martínez 3288* (MEXU), *L. Trejo s/n* (UAT); Ocampo, *T. W. Walters TW-201-11-A* (XAL), *TW-201-11-B* (MEXU); Tula, *D. W. Stevenson 569H, 569G, 569K* (MEXU), *F. Nicolalde-Morejón et al. 2357-2365* (CIB), *L. Martínez-Domínguez et al. 854-881* (CIB), *S. Avendaño 5328* (MEXU).

#### 8. Ceratozamia latifolia

Taxon classificationPlantaeCycadalesZamiaceae

Hort. Belg. ex Miq. Tijdschr. Wis-Natuurk. Wetensch. Eerste Kl. Kon. Ned. Inst. Wetensch. 1 (4): 206. 1848.

**Figure 10.**
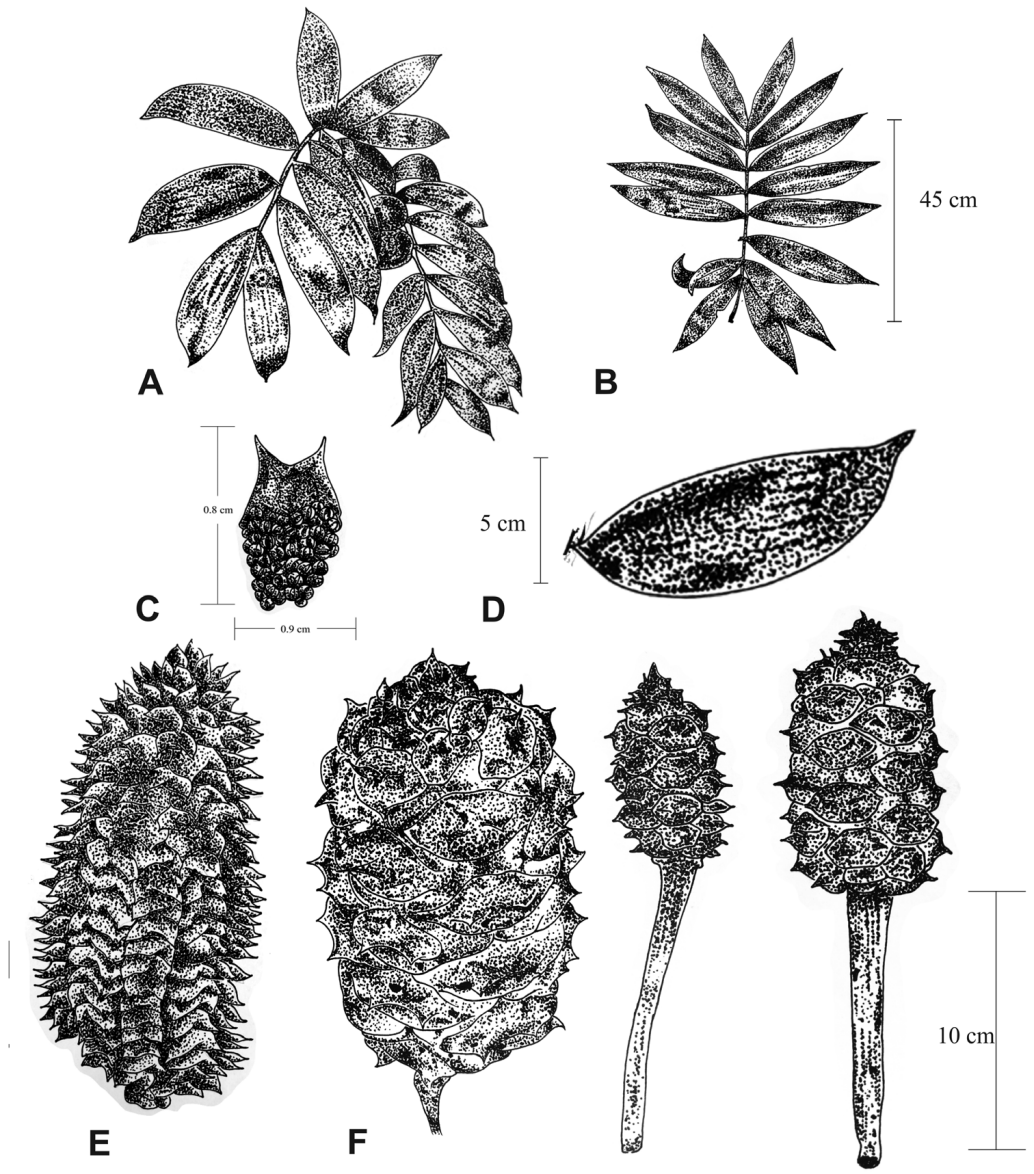
*Ceratozamia latifolia*.

Figures 3A , 5A , 10

##### Type.

MEXICO. San Luis Potosí: 20 Jul. 1983, (neotype, designated by Stevenson and Sabato 1986, *Stevenson 565 E* (NY); isoneotypes: MEXU).

Ceratozamia mexicana var. latifolia (Miquel) Schuster. Pflanzenr. 99:131. 1932. Type: Based on *Ceratozamia latifolia* Miq.

*Ceratozamia microstrobila* Vovides & Rees. Madroño, 30: 9–42. 1983. Type: MEXICO. San Luis Potosí, Ciudad del Maíz, 7 Nov. 1974, *J. Rees 1613* (holotype: XAL).

##### Description.

*Stem* semihypogeous, erect, 5–15 cm in length, 10–25 cm in diameter. *Cataphylls* persistent, densely tomentose at emergence, reddish-brown and partially tomentose at maturity, triangular, apex acuminate, 1.5–3 × 2–4 cm at base. *Leaves* 1–8, descending, 53–163.5 cm, reddish-brown at emergence, whitish-grey pubescence, glabrous at maturity. *Petiole* terete, straight, 25–80 cm, generally unarmed, greenish- brown in adult leaves. *Rachis* terete, straight, 25–110 cm, unarmed, greenish-brown in adult leaves. *Leaflets* 7–22, oblong, mostly planar, basally falcate, papyraceous, flat, opposite to subopposite, plane, green, adaxial and abaxial side glabrous, acuminate apex, asymmetric apex, attenuate at base, with conspicuous and indistinct veins; median leaflets 12–28 × 2.3–5.1 cm, 1.7–12.5 cm between leaflets; articulations brown, 0.4–1.1 cm wide. *Polliniferous strobilus* solitary, cylindrical, erect, 10.5–20 cm in length, 2.1–2.5 cm in diameter, greenish-yellow with reddish-brown pubescence at emergence, reddish-brown at maturity; peduncle tomentose, reddish-brown to brown, 5–11 cm in length, 0.9–1.2 cm in diameter; microsporophylls 0.5–1 × 0.6–1.1 cm, non-recurved distal face. *Ovuliferous strobilus* solitary, globose, erect, 7.5–16 cm in length, 5.5–7 cm in diameter, light green at emergence with brown trichomes, greyish-light brown with brown trichomes at maturity, apiculate apex; peduncle tomentose, brown, 4–13.5 cm in length, 1.5–1.8 cm in diameter; megasporophylls 24–56, 1.2–2 × 1.7–2.5 cm, prominent distal face, obtuse angle between horns. *Seeds* ovoid, sarcotesta whitish-red when immature, light brown at maturity, 1.5–2 cm in length, 1.2–1.5 cm in diameter.

##### Distribution and habitat.

This species is distributed widely in San Luis Potosí and southeast of Querétaro mountain region, between 600–1,100 m elevation (Fig. 9). The vegetation type of its habitat is pine-oak forest and cloud forest.

##### Etymology.

The specific epithet is derived from the Latin word for wide leaf (latus=wide and folium=leaf).

##### Distinguishing features.

Leaves reddish-brown at emergence, petiole generally unarmed, leaflets oblong, mostly planar, not basally falcate and papyraceous with asymmetric apex; ovulate strobilus greyish-light brown with brown trichomes at maturity, apiculate apex, prominent distal face and obtuse angle between horns (Fig. 5A).

##### Specimens examined.

MEXICO. **San Luis Potosí**: Ciudad del Maíz, *H. Puig 3420* (P), *J. Rees 1613* (MO, XAL), *S. Avendaño 5320* (MEXU), *S. Sabato et al. 2340* (MEXU, MO); El Naranjo, *D. W. Stevenson 567* (XAL), *F. Nicolalde-Morejón et al. 2375-2389* (CIB), *L. Martínez-Domínguez et al. 894-909* (CIB), *T. W. Walters TW-2001-08*, *TW-2001-10* (MEXU, XAL); Rayón, *A. P. Vovides et al. 1466* (XAL), *D. W. Stevenson 1117* (NY), *565 A* (MEXU), *565 B,C,E* (MEXU, XAL), F. *Medellin L. 1330* (MEXU), *27252* (ENCB), *S. Avendaño 5282* (MEXU); Tamasopo, A. *P. Vovides et al. 1465* (MEXU, XAL), *F. Medellin L. 27241* (ENCB), *F. Medellin L. s/n* (MEXU), *F. Nicolalde-Morejón et al. 2320-2348* (CIB), *L. Martínez-Domínguez et al. 811-844* (CIB).

##### Taxonomic comments.

As circumscribed here, *Ceratozamia latifolia* has been addressed in different ways, implying disagreements with the original Miquel (1848) description and, at the same time, bearing a relationship to the limits of *C. mexicana*. Several years after its original description, *C. latifolia* was considered doubtful (De Candolle 1864-1868); Miquel (1868-1869a, b) placed it in synonymy and as part of *C. mexicana*. On the other hand, Regel (1876a) considered it as a synonym of *C. miqueliana*, although ten years later Thiselton-Dyer (1884) recognised it as a species. However, Schuster (1983) transferred this binomial to the category of variety under the epithet C. mexicana var. latifolia, a status maintained until the work of Vovides et al. (1983). In our treatment, this binomial is recognised as a species, rendering it incongruent with C. mexicana var. latifolia sensu Vovides et al. (1983). This decision stems from the typification by Stevenson and Sabato (1986), where a neotype specimen geographically located in San Luis Potosí was designated to validate the binomial. In addition, we consider that *C. microstrobila* is part of *C. latifolia*, due to the absence of morphological and molecular evidence to recognise the former as a distinct species.

#### 9. Ceratozamia mexicana

Taxon classificationPlantaeCycadalesZamiaceae

Brongn. Ann. Sci. Nat., Bot. ser. 3, 5: 7–8, t. 1. 1846.

Figures 3L , 11

##### Type.

MEXICO, from Ghiesbrecht, cult. in Hort. Bot. Parisiensis, 1845, *Brongniart s.n.* (holotype: P). Epitype (designated by Vovides et al. 2016). MEXICO. Veracruz: Totutla, 08 Sep. 1982, *J. Rees & A. P. Vovides 1688* (XAL).

*Ceratozamia intermedia* Miq. Tijdschr. Wis-Natuurk. Wetensch. Eerste Kl. Kon. Ned. Inst. Wetensch. 1 (4): 40–41. 1848. Neotype (designated here). MEXICO. Veracruz: Puente Nacional, 13 Mar. 1985, *G. Castillo-Campo & Medina 4275* (XAL).

This specimen represents part of the variation found in *Ceratozamia mexicana* and the locality is on the collection route that was followed by 19^th^ century collectors to the centre of Veracruz.

*Ceratozamia longifolia*. Miq. Tijdschr. Wis-Natuurk. Wetensch. Eerste Kl. Kon. Ned. Inst. Wetensch. 1 (4): 40. 1848. Neotype (designated here). MEXICO. Veracruz: Zacuapam, Apr. 1913, *Purpus s/n* (MO [6362]).

This locality is a historic collection from the central region of Veracruz for *Ceratozamia mexicana*, which was one of the most collected cycads during the 19^th^ century.

Ceratozamia mexicana Brongn. var. mexicana.

*Ceratozamia longifolia* var. minor. Miq. Tijdschr. Wis-Natuurk. Wetensch. Eerste Kl. Kon. Ned. Inst. Wetensch. 2 (4): 290. 1849. Neotype (designated here). MEXICO. Veracruz: Teocelo, 23 Dic. 1975, *M. G. Zola 146* (XAL).

This locality is on the historic collection route for *Ceratozamia* and represents the northern end of the distribution for *Ceratozamia mexicana* where plants with lower leaflet widths have been recorded.

Ceratozamia mexicana var. longifolia. (Miquel) Dyer. Biol. cent.- amer., Bot. 3 (16): 193. 1884. Type: Based on *Ceratozamia longifolia*.

##### Description.

*Stem* epigeous, erect and decumbent, 20–80 cm in length, 20–45 cm in diameter. *Cataphylls* persistent, densely tomentose at emergence, reddish-brown and partially tomentose at maturity, triangular, apex acuminate, 2–6 × 2.5–6 cm at base. *Leaves* 5–55, descending, 100–270 cm, light green at emergence, brown pubescence, glabrous at maturity. *Petiole* terete, straight, 30–93 cm, armed with short and thin prickles, dark green in adult leaves. *Rachis* terete, straight, 56–154 cm, armed with prickles and unarmed, green in adult leaves. *Leaflets* 25–42, lanceolate, mostly planar, basally falcate, coriaceous, flat, opposite to subopposite, plane, green, adaxial and abaxial side glabrous, acuminate and symmetric apex, attenuate at base, with conspicuous and light green veins; median leaflets 29–51 × 2.3–3.7 cm, 1.8–4 cm between leaflets; articulations green, 0.6–1.5 cm wide. *Polliniferous strobilus* generally solitary (1–2), cylindrical, erect, 24–40 cm in length, 5.5–7.6 cm in diameter, greenish-yellow at emergence, greenish-yellow with blackish pubescence at maturity; peduncle tomentose, reddish-brown to light brown, 3.5–5 cm in length, 1.8–2.3 cm in diameter; microsporophylls 1.6–2.7 × 0.9–2.1 cm, non-recurved distal face. *Ovuliferous strobilus* generally solitary (1–2), cylindrical, erect or pendular, 23.5–38 cm in length, 10.5–14.6 cm in diameter, dark green with blackish pubescence at emergence, dark green with black and grey trichomes at maturity, acuminate apex; peduncle tomentose, brown to reddish-brown, 8–11.5 cm in length, 2.5–3 cm in diameter; megasporophylls 55–224, 2–4 × 4.4–5.3 cm, prominent distal face, obtuse angle between horns. *Seeds* ovoid, sarcotesta whitish-yellow to yellow when immature, light brown at maturity, 2–3.3 cm in length, 1.5–2.5 cm in diameter.

##### Distribution and habitat.

Endemic to Mexico from the River La Antigua drainage system within Xico, Teocelo and Coatepec municipalities to south end of Sierra Madre Oriental in Veracruz state, between 500–1,300 m in cloud forest (Figure 8).

##### Etymology.

The epithet is derived from the country of origin of the material for the description of the genus.

##### Distinguishing features.

**Figure 11.**
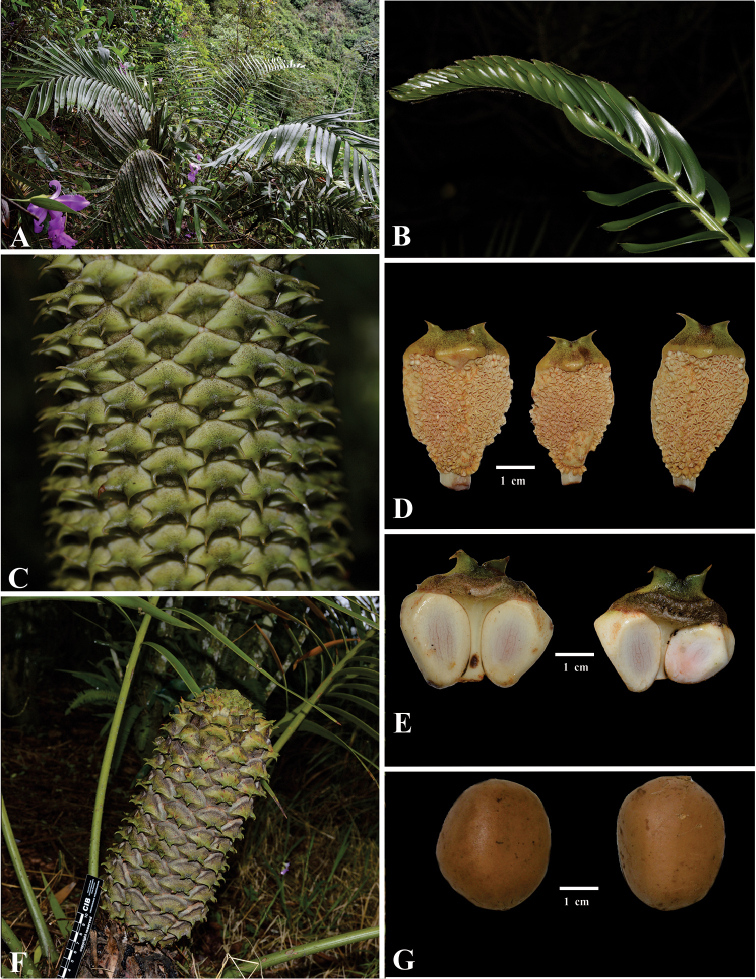
*Ceratozamia mexicana*. **A** Adult plant in habit **B** Leaf at emergence **C** Detail of pollen strobili **D** Microsporophyll **E** Megasporophyll **F** Detail of ovulate strobili **G** Seeds.

*Ceratozamia mexicana* is distinguished by its lanceolate, coriaceous and flat leaflets with a symmetric apex, ovulate strobilus dark green with black and grey trichomes at maturity, and a prominent distal face with an obtuse angle between horns (Fig. 11).

##### Specimens examined.

MEXICO. **Veracruz**: Coatepec, *P. Zamora C. 2450* (MEXU, XAL); Comapa, *F. Nicolalde-Morejón et al. 2146-2156* (CIB), *L. Martínez-Domínguez et al. 164, 716-730* (CIB); Puente Nacional, *G. Castillo-Campos & M. E. Medina 4299* (XAL); Sochiapa, *M. Vázquez-Torres 8589* (CIB); Teocelo, *F. Nicolalde-Morejón et al. 2273-2278* (CIB), *M. G. Zola 146* (XAL), *M. Vázquez-Torres* 4865 (CIB), *L. Martínez-Domínguez et al. 731-750, 764-770* (CIB); Tlaltetela, *F. Nicolalde-Morejón & L. Martínez-Domínguez 2246, 2253-2256* (CIB), *L. Martínez-Domínguez & F. Nicolalde-Morejón 584-586, 620, 628-635* (CIB); Totutla, *A. P. Vovides 730-733, 748* (XAL), *Brigada T. Walters s/n* (XAL), *F. Nicolalde-Morejón et al. 2279-2281* (CIB), *F. Vázquez B. 730* (XAL), *J. Rees 6344* (IEB), *J. Rees & A. P. Vovides 1660, 1672, 1689* (XAL), *L. Martínez-Domínguez et al. 704-707, L. Martínez-Domínguez et al. 752-758* (CIB); Xico, *L. Martínez-Domínguez & F. Nicolalde-Morejón 640-648* (CIB).

##### Taxonomic comments.

The taxonomy of *Ceratozamia mexicana* has undergone constant changes since its original publication in 1846. The identity of this binomial was ambiguous for several decades. This taxonomic uncertainty was due to the complexity of relationships with some of the other names published by Miquel during the immediately succeeding years (1847, 1848 and 1849), following the original publication of the genus –namely, *C. brevifrons*, *C. robusta*, *C. intermedia*, *C. longifolia*, and *C. latifolia*. Later on, some names were placed in synonymy and others were characterised as *nomina nuda*; in particular, *C. brevifrons* was listed as a synonym of *C. mexicana* under the assumption that it was in fact a juvenile form of the latter (see Miquel 1848, 1861).

In further taxonomic treatments of *Ceratozamia* –specifically, in De Candolle (1864–1868) and including Miquel’s *Nouveaux matériaux pour servir à la connaissance des Cycadées* (1868–1869a, b)– this nomenclatural status was maintained. In his mature work, Miquel considered the variation observed between juveniles and adults was due to the corresponding ontogenetic modifications and collapsed into synonymy with *C. mexicana* all five species previously described.

Half a century later, Schuster (1932) circumscribed the genus to include only two species: *Ceratozamia kuesteriana* and *C. mexicana*, with two varieties and two forms. However, in the first modern taxonomic treatment (Vovides et al. 1983) and continuing with the typification of names within the genus (Stevenson and Sabato 1986), the type species was circumscribed to the central region of Veracruz. In this context, Vovides et al. (1983) attributed a wide geographic distribution and high variation of morphological characters to *C. mexicana* while recognising three varieties: C. mexicana var. mexicana, C. mexicana var. latifolia (Miq.) Schuster and C. mexicana var. robusta (Miq.) Dyer.

The original *Ceratozamia mexicana* specimens were collected by the Belgian botanist and explorer A. B. Ghiesbreght, who probably did his fieldwork in the region of Huatusco, Veracruz. This location is recognised as part of his route for botanical collections between 1835 and 1838 (Barnhart 1965; Sartorius 1990; Rzedowski et al. 2009). Besides the “El Mirador” locality, mentioned by Thiselton-Dyer (1884) and Schuster (1932) as the main reference place, the species still exists in Huatusco. Due to coffee cultivation, the original vegetation has been replaced almost in its entirety. However, the species still occurs in the wild in relictual secondary vegetation within a property called “Hacienda Zacuapam”. This property was part of a former “El Mirador” hacienda, early in the XIX century (Sartorius 1990).

#### 10. Ceratozamia morettii

Taxon classificationPlantaeCycadalesZamiaceae

Vázq. Torres & Vovides. Novon 8 (1): 87–90. 1998.

Figure 3C

##### Type.

MEXICO. Veracruz: Landero y Coss, 7 Jan. 1992, *M. Vázquez-Torres & H. Barney 4097* (holotype: CIB; isotypes: CIB, MEXU, XAL).

##### Description.

*Stem* epigeous, erect and decumbent, 20–50 cm in length, 20–35 cm in diameter. *Cataphylls* persistent, densely tomentose at emergence, reddish-brown and partially tomentose at maturity, triangular, apex acuminate, 4–6.5 × 3–3.5 cm at base. *Leaves* 3–30, descending, 82–200 cm, light green at emergence, brown pubescence, glabrous at maturity. *Petiole* terete, straight, 30–90 cm, armed with short and thin prickles, green in adult leaves. *Rachis* terete, straight, 50–116 cm, armed with prickles, green in adult leaves. *Leaflets* 10–23, oblong, planar and abaxially curved, not basally falcate, coriaceous, flat, opposite to subopposite, plane, green, adaxial side glabrous and abaxial side glaucous, acuminate apex, asymmetric apex, attenuate at base, with conspicuous and light green veins; median leaflets 17.5–41 × 2.6–4.3 cm, 2.1–6 cm between leaflets; articulations green and yellow, 0.5–1.6 cm wide. *Polliniferous strobilus* solitary, cylindrical, erect, 12–15 cm in length, 3.8–4.5 cm in diameter, brownish-yellow at emergence, greenish-yellow with brown pubescence at maturity; peduncle tomentose, reddish-brown to brown, 3–7 cm in length, 1.1–1.3 cm in diameter; microsporophylls 1.0–1.3 × 0.8–1 cm, non-recurved distal face. *Ovuliferous strobilus* solitary, globose, erect, 14–18 cm in length, 8–9.5 cm in diameter, yellowish-green with brown pubescence at emergence, green with blackish trichomes at maturity, apiculate apex; peduncle tomentose, brown, 4–7 cm in length, 1.2–1.8 cm in diameter; megasporophylls 40–108, 2.1–2.6× 3.2–3.6 cm, truncate distal face, right angle of horns. *Seeds* ovoid, sarcotesta whitish-yellow to yellow when immature, light brown at maturity, 1.5–2 cm in length, 1–1.2 cm in diameter.

##### Distribution and habitat.

Endemic to Veracruz, on karstic rocks and cliffs of the Sierra de Chiconquiaco, between 1,200–1,850 m (Fig. 8). The vegetation type where this species grows is cloud forest.

##### Etymology.

The specific epithet honours Aldo Moretti, in recognition of his scientific contributions in the field of cycad biology. He is a researcher in the Orto Botanico and Istituto di Biologia Vegetale at the University of Naples Federico II, Italia.

##### Distinguishing features.

*Ceratozamia morettii* is similar to *C. delucana* in leaf morphology; however, there are differences in the total size of plant and in reproductive structures. This species has leaves with up to 23 pairs of leaflets and the ovulate strobilus is green with blackish trichomes at maturity, 14–18 cm long, 8–9.5 cm in diameter and an apiculate apex. Moreover, *C. morettii* has minimal variation at the population level.

##### Specimens examined.

MEXICO. **Veracruz**: Chiconquiaco, *A. P. Vovides 687, 704* (XAL), *C. J. W. Schiede s/n* (XAL), *J. Rees 6336* (IEB), *L. Lagunes G. 83, 84* (CIB); Landero y Coss, *A. P. Vovides 1662* (XAL), *J. Rees 6342* (IEB), *J. Rees & A. P. Vovides 1663, 1664, 1676* (XAL), *L. Martínez-Domínguez et al. 185-214* (CIB), *M. Vázquez-Torres 4097* (XAL), *8349* (CIB), *S. Avendaño 5378* (XAL), *T. W. Walters 2001-01-E* (XAL); Tenochtitlán, *A. Rincón G. et al. 296-298* (XAL); Yecuatla, *C. Gutiérrez B. 134* (XAL), *J. Rees 1677* (XAL), *F. Nicolalde-Morejón & L. Martínez-Domínguez 2087-2106* (CIB), *L. Martínez-Domínguez et al. 161* (CIB).

#### 11. Ceratozamia sabatoi

Taxon classificationPlantaeCycadalesZamiaceae

Vovides & Vázq. Torres. Novon 3 (4): 502–504. 1993.

Figures 2B , 3D , 6A , 7A

##### Type.

MEXICO. Querétaro: San Joaquín, 15 Apr. 1991, *A. P. Vovides 1205* (holotype: XAL).

##### Description.

*Stem* epigeous, erect and decumbent, 8–30 cm in length, 20–35 cm in diameter. *Cataphylls* persistent, densely tomentose at emergence, reddish-brown and partially tomentose at maturity, triangular, apex acuminate, 3–4.5 × 2–3.5 cm at base. *Leaves* 3–40, descending, 60–129 cm, dark brown at emergence, whitish-grey pubescence, glabrous at maturity. *Petiole* terete, straight, 20–60 cm, armed with short and thin prickles, greenish-brown in adult leaves. *Rachis* terete, straight, 40–92 cm, armed with prickles, brown in adult leaves. *Leaflets* 26–54, linear, planar and abaxially curved, basally falcate, papyraceous, flat, opposite to subopposite, plane, green, adaxial and abaxial side glabrous, acuminate apex, symmetric apex, attenuate at base, with conspicuous and light green veins; median leaflets 13–32 × 0.6–1.5 cm, 0.5–1.5 cm between leaflets; articulations brown, 0.3–0.7 cm wide. *Polliniferous strobilus* solitary, cylindrical, erect, 11–18 cm in length, 3.5–4.8 cm in diameter, greenish-yellow at emergence, greenish-yellow with blackish pubescence at maturity; peduncle tomentose, reddish-brown to brown, 7–10.5 cm in length, 1.1–1.9 cm in diameter; microsporophylls 1–1.9 × 1–1.4 cm, recurved distal face. *Ovuliferous strobilus* solitary, cylindrical, erect, 15.5–18 cm in length, 6–8.5 cm in diameter, yellowish-green with brown pubescence at emergence, blue green with blackish trichomes at maturity, apiculate apex; peduncle tomentose, brown, 4–7 cm in length, 1.2–1.8 cm in diameter; megasporophylls 98–108, 2.3–2.5× 2–3 cm, truncate distal face, right angle between horns. *Seeds* ovoid, sarcotesta whitish-red when immature, light brown at maturity, 1.2–2 cm in length, 1.2–1.5 cm in diameter.

##### Distribution and habitat.

Endemic to Mexico and known from the states of Querétaro and Hidalgo, at 1,600–1,900 m in the Sierra Gorda mountain range of Querétaro, along the mountain range northwest of Hidalgo (Fig. 9). It inhabits the understorey herbaceous layer of the transition zone between oak forest and cloud forest.

##### Etymology.

The specific epithet honours the late Sergio Sabato, distinguished professor at the University of Naples Federico II, Italia, for his contributions to knowledge of the biology and systematics of cycads, particularly in the Neotropics.

##### Distinguishing features.

Leaflets lanceolate, papyraceous, symmetric apex and with brown articulations; pollen strobilus greenish-yellow with blackish pubescence at maturity and microsporophylls with recurved distal face (Fig. 6A).

##### Specimens examined.

MEXICO. **Hidalgo**: Zimapán, *R. Contreras-Medina 55, 56* (XAL), *R. Fernandez-Nava 6561* (MEXU, MO, XAL). **Querétaro**: Cadereyta de Montes, *A. P. Vovides 1193, 1196-1199, 1201, 1205* (XAL), *A. P. Vovides et al. 1203* (MEXU, XAL), *F. Nicolalde-Morejón et al. 2169, 2170* (CIB), *L. Martínez-Domínguez et al. 313-343* (CIB), *O. V. Zirahuen 128014* (IEB), *R. Fernandez-Nava s/n* (QMEX), *R. Zirahuén-Ortega V. 328* (MEXU); Landa de Matamoros, *T. W. Walters 2001-05-A, B* (XAL); Pinal de Amoles, *F. Nicolalde-Morejón et al. 2171, 2172* (CIB), *L. Martínez-Domínguez et al. 344-372* (CIB), *Rzedowski s/n* (XAL).

#### 12. Ceratozamia tenuis

Taxon classificationPlantaeCycadalesZamiaceae

(Dyer) D. W. Stev. & Vovides. Botanical Sciences 94 (2): 419–429. 2016.

Figures 2A , 3I

##### Type.

Hort. Kew Palm House: *Thistleton-Dyer s.n. 1881* (lectotype: K). Epitype (designated here). MEXICO. Veracruz: Jilotepec, 19 Jan. 1976, *A. P. Vovides 18* (XAL). Type: Based on Ceratozamia mexicana var. tenuis Dyer Biol. Cent.-Amer., Bot. 3: 193. 1884. Isoepitype (designated here). *A. P. Vovides 18* (NY).

Ceratozamia mexicana var. vulgaris. Schuster. Pflanzener 99: 131. 1932. Neotype (designated here). MEXICO. Veracruz: Xalapa, Chiltoyac, 18 Oct. 2016, *Martínez-Domínguez et al. 984* (CIB).

Schuster mentioned “Jalapa” in his treatment and Chiltoyac (Xalapa, Veracruz), which is very near to Xalapa and thus seems appropriate because the plants match the description by Schuster.

Ceratozamia mexicana var. longifolia f. tenuis (Dyer) Schuster. Pflanzener 99: 132. 1932. Type: Based on Ceratozamia mexicana var. tenuis.

##### Description.

*Stem* epigeous, erect and decumbent, 20–100 cm in length, 30–45 cm in diameter. *Cataphylls* persistent, densely tomentose at emergence, reddish-brown and partially tomentose at maturity, triangular, apex acuminate, 2–6 × 2–5.5 cm at base. *Leaves* 6–56, ascending, 85–225 cm, dark green at emergence, brown pubescence, glabrous at maturity. *Petiole* terete, straight, 30–93 cm, armed with short and thin prickles, green in adult leaves. *Rachis* terete, straight, 56–154 cm, armed with prickles, green in adult leaves. *Leaflets* 30–56, linear, planar and abaxially curved, basally falcate, papyraceous, caniculate, opposite to subopposite, plane, green, adaxial and abaxial side glabrous, acuminate and symmetric apex, attenuate at base, with conspicuous and light green veins; median leaflets 23–50.5 × 1–2.1 cm, 0.3–2.5 cm between leaflets; articulations green, 0.4–1.4 cm wide. *Polliniferous strobilus* solitary, cylindrical, erect, 26–50 cm in length, 5–7 cm in diameter, greenish-yellow at emergence, greenish-yellow with blackish pubescence at maturity; peduncle tomentose, reddish-brown to light-brown, 3.7–22 cm in length, 1.2–2.5 cm in diameter; microsporophylls 1.7–2.7 × 1.2–1.9 cm, non-recurved distal face. *Ovuliferous strobilus* solitary, cylindrical, erect or pendular, 22–35 cm in length, 7.6–13.3 cm in diameter, dark green with blackish pubescence at emergence, dark green with blackish trichomes at maturity, acuminate apex; peduncle tomentose, brown to reddish-brown, 8–16.5 cm in length, 1.5–2.4 cm in diameter; megasporophylls 48–195, 2.7–3.1 × 4.2–5 cm, prominent distal face, right angle between horns. *Seeds* ovoid, sarcotesta whitish-yellow to yellow when immature, light brown at maturity, 2.5–3 cm in length, 1.3–1.8 cm in diameter.

##### Distribution and habitat.

Endemic to Mexico in the central Veracruz mountain region at 1,200–1,850 m elevation on volcanic soils with basaltic rocks (Fig. 8). The vegetation type of the habitat is cloud forest.

##### Etymology.

The specific epithet alludes to thin leaflets.

##### Distinguishing features.

Characterised by leaflets linear, papyraceous and caniculate with symmetric apex; petiole and rachis armed with thin prickles; new leaves are dark green at emergence; ovulate strobilus dark green with blackish trichomes at maturity, prominent distal face, and right angle between horns.

##### Specimens examined.

MEXICO. **Veracruz**: Chiconquiaco, *D. Jimeno Sevilla 754* (XAL), *J. Rees 1625* (XAL), *1626* (MEXU, XAL), *L. Martínez-Domínguez et al. 971-981* (CIB), *R. Fernandez-Nava 385* (MEXU); Coacoatzintla, *F. Vazquez B*. (XAL), G. *Castillo-Campos 118* (XAL), *L. Martínez-Domínguez et al. 165-184*, 273-282, 759 (CIB); Jilotepec, *A. P. Vovides 470* (XAL), *471* (MEXU, XAL), *735* (XAL), *E. Estrada et al. 757* (MEXU), *J. Rees 1620* (XAL), *F. Nicolalde-Morejón* & *L. Martínez-Domínguez 2067-2086* (CIB), *L. Martínez-Domínguez et al. 573-583* (CIB), *M. G. Zola 667* (MEXU, XAL), *R. Ortega J. 525* (XAL), *S. Avendaño 5395* (MEXU); Tepetlán, *F. Nicolalde-Morejón & L. Martínez-Domínguez 2001-2004* (CIB), *2047-2066* (CIB), *2217-2226* (CIB), *L. Martínez-Domínguez et al. 160, 283-293, 545-555* (CIB); Xalapa, *L. Martínez-Domínguez et al. 985-987* (CIB).

##### Taxonomic comments.

This name has been controversial due to a series of transferences and the lack of material. This species was initially described as C. mexicana var. tenuis (Thiselton-Dyer 1884) and Schuster (1932) transferred it to form status under the name C. mexicana var. longifolia f. tenuis. However, its identity has been questioned for decades due to the scarcity of type specimens. The discovery of a specimen collected and examined by Thiselton-Dyer for his protologue description and the subsequent lectotypification of this specimen, has allowed a clearer concept of this taxon (Vovides et al. 2016). Recently, this entity was transferred to the species level after the analysis of quantitative morphological and anatomical evidence which allowed the separation of populations previously associated with the *C. mexicana* and their assignment as a new entity (Vovides et al. 2016). Individuals in these populations bear correspondence to lectotype specimens in the Royal Botanic Gardens Kew herbarium, which correspond to Ceratozamia mexicana var. tenuis (Vovides et al. 2016). Two specimens were incorrectly designated by Vovides et al. (2016) as epitypes [A. Vovides 018 (XAL, NY)]. According to the International Code of Nomenclature (McNeil et al. 2012), we correct the designation of the epitype and designate an isoepitype (Section 2, Article 9, Recommendation 9C). Finally, we note that the historical *Ceratozamia* populations from Jilotepec and Coacoatzintla have been associated to the *C. mexicana* species since the morphological work of Chamberlain (1912).

#### 13. Ceratozamia totonacorum

Taxon classificationPlantaeCycadalesZamiaceae

Mart.-Domínguez & Nic.-Mor. Brittonia. 2017.

Figure 3J

##### Type.

MEXICO. Puebla: Jonotla, 9 Jun. 2015, *L. Martínez-Domínguez* & *F. Nicolalde-Morejón 618* ♀ (holotype: CIB).

##### Description.

*Stem* epigeous, erect and decumbent, 10–45 cm in length, 10–25 cm in diameter. *Cataphylls* persistent, densely tomentose at emergence, reddish-brown and glabrous at maturity, triangular, apex acuminate, 2–5 × 1.2–2.5 cm at base. *Leaves* 10–55, descending, 90–265 cm, brown at emergence, with brown pubescence, glabrous at maturity. *Petiole* terete, straight, 30–70 cm, armed with long and thin prickles, green in adult leaves. *Rachis* terete, straight, 85–181 cm, armed with prickles, green in adult leaves. *Leaflets* 11–33, oblong, mostly planar, not basally falcate and occasionally falcate, papyraceous, flat, opposite to subopposite, plane, green, adaxial side glabrous and glaucous, abaxial side glaucous, acuminate apex, symmetric apex, attenuate at base, with conspicuous and light green veins; median leaflets 17–40 × 2.7–4.2 cm, 2–5.6 cm between leaflets; articulations green, 0.5–1.3 cm wide. *Polliniferous strobilus* generally solitary (1–2), cylindrical, erect, 29–31 cm in length, 5.4–5.6 cm in diameter, greenish- yellow at emergence, yellow with brown pubescence at maturity; peduncle tomentose, light brown, 9–12 cm in length, 1.5–2 cm in diameter; microsporophylls 1.5–2.2 × 1.3–1.8 cm, non-recurved distal face. *Ovuliferous strobilus* solitary, cylindrical, erect or pendular, 20.5–26 cm in length, 8.5–9.3 cm in diameter, light green and glaucous, with orange to light brown pubescence at emergence, green with yellowish-brown trichomes at maturity, acuminate apex; peduncle tomentose, light brown, 10–11.2 cm in length, 1.3–1.5 cm in diameter; megasporophylls 74–92, 1.6–2.2 × 2.6–3.4 cm, prominent distal face, right angle between horns. *Seeds* ovoid, sarcotesta whitish-red when immature, cream to light brown at maturity; 3.3–4.0 cm in length, 0.8–1.2 cm in diameter.

##### Distribution and habitat.

Endemic to Mexico in the Sierra Norte of Puebla mountain region on rocky outcrops in exposed walls up to 80 m at 600−1,050 m (Fig. 8). It inhabits the transition zone between cloud forest and evergreen tropical forest.

##### Etymology.

The specific epithet makes reference to the Totonaco ethnic group of Santiago Ecatlán in Sierra Norte of Puebla, whose residents use and manage this species in local cultural contexts related to rituals.

##### Distinguishing features.

This species is distinguished by its petioles with abundant and long, thin prickles, brown leaves at emergence, but the colour disappears in the adult leaves; leaflets are oblong and papyraceous with asymmetric apex. Ovulate strobilus yellowish-green with brown trichomes.

##### Specimens examined.

MEXICO. **Hidalgo**: Huehuetla, *A. P. Vovides 23* (XAL). **Puebla**: Jonotla, *F. Nicolalde-Morejón et al. 1948* ♂ (CIB), *1956* (MEXU), *1957* (NY), *1949-1955* (CIB), *1958-1967* (CIB), *F. Nicolalde-Morejón et al. 1966* (CIB), *L. Martínez-Domínguez & F. Nicolalde-Morejón 619* ♀ (CIB); Pahuatlán, *G. Toriz et al. 226* (MEXU); Tlapacoyan, *E. Meza P. 14* (XAL). **Veracruz**: Tlachichilco, *A. Rincón G. et al. 2584* (XAL), *2585* (MEXU, XAL).

#### 14. Ceratozamia zaragozae

Taxon classificationPlantaeCycadalesZamiaceae

Medellín-Leal. Brittonia 15: 175–176. 1963.

Figures 4B , 5C

##### Type.

MEXICO. San Luis Potosí: Río Verde, 22 Jul. 1962. *F. Medellín-Leal 1452* (holotype: SLPM; isotypes: ENCB, MICH, US).

##### Description.

*Stem* semihypogeous, erect, 10–20 cm in length, 10–15 cm in diameter. *Cataphylls* persistent, densely tomentose at emergence, reddish-brown and partially tomentose at maturity, triangular, apex acuminate, 1.8–2.5 × 1–2 cm at base. *Leaves* 3–27, ascending, 95–202 cm, reddish-brown at emergence with whitish-grey pubescence, glabrous at maturity. *Petiole* terete, twisted, 11–36 cm, unarmed, green in adult leaves. *Rachis* terete, twisted, 40–77 cm, unarmed, green in adult leaves. *Leaflets* 25–46, linear, mostly planar, basally falcate, membranaceous, strongly caniculate, opposite to subopposite, plane, green, adaxial and abaxial side glabrous, acute apex, symmetric apex, attenuate at base, with conspicuous and light green veins; median leaflets 17–31.5 × 0.4–0.7 cm, 0.8–2.3 cm between leaflets; articulations yellow, 0.2–0.3 cm wide. *Polliniferous strobilus* solitary, cylindrical, erect, 15–19 cm in length, 2–3.5 cm in diameter, greenish with reddish-brown pubescence at emergence, reddish-brown at maturity; peduncle tomentose, reddish-brown to brown, 5–8 cm in length, 1.5–1.8 cm in diameter; microsporophylls 0.8–1.2 × 0.3–0.6 cm, non-recurved distal face. *Ovuliferous strobilus* solitary, cylindrical, erect, 8.2–12 cm in length, 5.8–7 cm in diameter, green with scarcely reddish-brown trichomes at emergence, dark green at maturity, acute apex; peduncle tomentose, brown, 6–9 cm in length, 0.9–1.2 cm in diameter; megasporophylls 24–49, 2.2–3.7 × 2–2.6 cm, truncate distal face, obtuse angle between horns. *Seeds* ovoid, sarcotesta light brown at maturity, 2–2.8 cm in length, 1.8–2 cm in diameter.

##### Distribution and habitat.

Endemic to Mexico in a small mountain range in South Central San Luis Potosí at 1,500–1,800 m elevation (Fig. 9). The vegetation type of the habitat is pine-oak forest.

##### Etymology.

The specific epithet is refers to General Ignacio Zaragoza, a hero of the Battle of Puebla (May 1862) against the French Army.

##### Distinguishing features.

This species is distinguished by having petiole and rachis unarmed and twisted. Leaflets are lanceolate, mostly planar, not basally falcate, membranaceous and caniculate, with symmetric apex.

##### Specimens examined.

MEXICO. **San Luis Potosí**: Río Verde, *A. G. Mendoza & L. Vargas 1389* (MEXU), *A. P. Vovides 435* (XAL), *E. Molseed 34* (MEXU), *F. Nicolalde-Morejón et al. 2307-2319* (CIB), *L. Martínez-Domínguez et al. 792-808* (CIB), T. *Walters et al. TW-2001-07* (MEXU, XAL).

## Discussion

The taxonomic history of species names in the genus Ceratozamia, as presented here, allow us to conclude that infrageneric concepts in this gymnosperm taxon are increasingly stringent and that infraspecific ranks are no longer recognised (cf. Osborne and Walters 2004). Most of these changes elevate varieties through the inclusion of new species. In general, several taxonomic changes have taken place, including diverse transfers at the species level, and substantial taxonomic disagreements have occurred, such as those involving the synonymy of *C. microstrobila*. In particular, the case of *C. mexicana* has been controversial, as demonstrated by the series of relationships and taxonomic changes, which have been established around it. With respect to the recent publication of two independent works in which contrasting taxonomic scenarios were proposed with respect to *C. mexicana* (Medina-Villareal and González-Astorga 2016; Vovides et al. 2016), the present morphological evaluation validates the proposal of Vovides et al. (2016).

Further support can be raised in connection with our position on recent taxonomic stances for *Ceratozamia mexicana*. On the basis of their phenetic analysis, Medina-Villarreal and González-Astorga (2016) suggested that the type species of the genus, *C. mexicana*, should be geographically anchored to the locality of the *C. brevifrons* neotype, given its morphometric similarity to the *C. mexicana* holotype. Under this assumption, these authors consider that *C. decumbens*, *C. morettii* and *C. brevifrons* should all be synonyms of *C. mexicana*. On the other hand, according to Medina-Villarreal and González-Astorga (2016), populations from the Naolinco Valley, Veracruz, should be described as a new species. Vovides et al. (2016) disagree on this point and suggest that those populations correspond in turn to C. mexicana var. tenuis, whose taxonomic treatment would then be recognised at the species level as *C. tenuis*. Under this perspective, *C. mexicana* would be recircumscribed to populations in the southern extreme of Veracruz. With our recircumscription proposal and review of historical documents, we consider that the locality of the *C. mexicana* holotype corresponds to the proposal of Vovides et al. (2016).

## Supplementary Material

XML Treatment for Ceratozamia brevifrons

XML Treatment for Ceratozamia chamberlainii

XML Treatment for Ceratozamia decumbens

XML Treatment for Ceratozamia delucana

XML Treatment for Ceratozamia fuscoviridis

XML Treatment for Ceratozamia hildae

XML Treatment for Ceratozamia kuesteriana

XML Treatment for Ceratozamia latifolia

XML Treatment for Ceratozamia mexicana

XML Treatment for Ceratozamia morettii

XML Treatment for Ceratozamia sabatoi

XML Treatment for Ceratozamia tenuis

XML Treatment for Ceratozamia totonacorum

XML Treatment for Ceratozamia zaragozae
